# VCFedAdam: A Verifiable and Compressed FedAdam Aggregation Scheme for Federated Learning in Distributed Sensor Networks †

**DOI:** 10.3390/s26185814

**Published:** 2026-09-14

**Authors:** Zhiwei Si, Xiuheng Liao, Ziang Wu, Tianhui Li, Chunhua Su

**Affiliations:** Graduate School of Computer Science and Engineering, University of Aizu, Aizu-Wakamatsu 965-8580, Japan; d8272107@u-aizu.ac.jp (Z.S.); d8262105@u-aizu.ac.jp (Z.W.); lith@u-aizu.ac.jp (T.L.); chsu@u-aizu.ac.jp (C.S.)

**Keywords:** federated learning, privacy-preserving, sketch compression, verifiable aggregation, sensor-network

## Abstract

Federated learning enables collaborative training across distributed sensor networks (DSNs) without requiring the sharing of raw sensing data. However, local updates can leak private information, and untrusted servers may return incorrect aggregation results. Existing verifiable secure aggregation schemes often incur high costs for resource-constrained devices. To address this, a verifiable and compressed FedAdam aggregation scheme (VCFedAdam) is proposesed. VCFedAdam performs masking, aggregation and verification operations within a low-dimensional sketch space, with the server recovering only the aggregated result for a fixed online set *R*, which consists of the users that successfully submit valid masked sketches in the current round and is fixed before aggregate recovery. Additionally, a commitment-bound verification (CBV) mechanism is designed to prevent the server from adaptively tampering with the aggregated results. At a compression ratio of 25%, experimental results show that VCFedAdam reduces computation overhead by 90.25% and communication overhead by 75.38% compared with traditional secure aggregation. On the CIFAR-10 and MNIST datasets, accuracy drops by only 0.64% and 1.16%, respectively. Furthermore, security analysis confirms that VCFedAdam achieves both privacy protection and aggregation verifiability.

## 1. Introduction

Distributed sensor networks (DSNs) are widely used in applications such as wearable healthcare, intelligent transportation, industrial monitoring, and environmental sensing [1]. A large number of sensor devices continuously collect local data. These data are usually distributed across different devices and may contain sensitive information about user behavior, locations, physical conditions, or industrial processes. Uploading all data to a central server can increase communication overhead and introduce privacy risks [2,3]. Therefore, DSNs require a collaborative learning approach that can utilize distributed data without directly sharing raw data [4].

Federated learning (FL), as a collaborative learning paradigm for distributed data, naturally meets this requirement [3]. FL allows sensor devices to train models locally and upload only model updates for global aggregation. This manner reduces the need to transmit raw sensing data and makes FL well suited to distributed sensing environments [5]. However, model updates may still reveal sensitive information about the underlying training data [6]. The aggregation server also cannot be fully trusted. It may attempt to infer information about individual users or return incorrect aggregation results. Therefore, FL in DSNs requires both privacy protection for local updates and verification of the aggregation results returned by the server [7].

Existing privacy-preserving FL schemes commonly employ secure aggregation (SA) [8], homomorphic encryption (HE) [9], secret sharing (SS) [10], and differential privacy (DP) [11]. Verifiable aggregation further uses homomorphic hashes [12], commitments [13], message authentication codes [14], or auxiliary entities to check aggregation results. These methods improve privacy and integrity, but most still perform security operations in the original model space. Masking, aggregate recovery, and verification are usually related to the model dimension *d*. As the model size increases, these security costs also increase. Sensor devices usually have limited bandwidth, computation capability, and energy [3]. Therefore, high-dimensional security operations can impose a significant burden on such devices. Model compression can reduce transmission overhead, but simply adding compression before an existing secure aggregation protocol is insufficient. The compressed representation still contains information about user updates and therefore must also be protected and verified [15].

**Motivation.** Existing verifiable aggregation schemes improve privacy and integrity, but most still operate on full-dimensional model updates. VerifyNet [16], EVSA [17] and VCFL [13] reduce different parts of the aggregation cost, but their masking, recovery, or verification still depend on the original model dimension. This creates high overhead for resource-constrained sensor devices. Sketch compression can reduce this cost, but compressed updates still require privacy protection and result verification [18]. Therefore, a lightweight scheme is needed to perform secure aggregation and verification directly in the sketch domain while keeping aggregate recovery bound to a fixed online set *R*.

To address above problem, we propose VCFedAdam to perform secure aggregation directly in a low-dimensional sketch space instead of the original model space. Each individual sketch remains protected, while the server can recover only the aggregate sketch for the fixed online set *R*. The same set *R* is also used in subsequent verification. This prevents the server from isolating an individual contribution through aggregate results obtained from different user subsets. We further design Commitment-Bound Verification (CBV). The server must first fix the current aggregation state before receiving fresh audit information. Therefore, the subsequent verification always refers to a predetermined aggregation object. The server cannot modify the result to be verified after obtaining the audit information.

VCFedAdam also distinguishes aggregation availability from verification status. Only a result that completes CBV is regarded as verified. If a user drops out during the subsequent audit phase, the result is marked as unverified and does not obtain the integrity guarantee provided by CBV. For a verified aggregate sketch, the server performs sketch decoding and applies FedAdam to update the global model. Local error feedback is used to compensate for the approximation error introduced by compression. In this way, VCFedAdam forms a complete framework that integrates low-dimensional secure aggregation, set-consistent aggregate recovery, commitment-bound verification, error compensation, and adaptive optimization.

An earlier version of VCFedAdam has been accepted for publication in the proceedings of AIBlock’2026 [19]. The present article substantially extends the conference version by adapting VCFedAdam to DSNs, introducing CBV, strengthening the security analysis under an extended threat model, and providing a more comprehensive performance and model-utility evaluation.

The main contributions of this work are summarized as follows.

- A verifiable and compressed FedAdam aggregation scheme for FL (VCFedAdam) is proposed. VCFedAdam moves masking and aggregation from the original model space to a low-dimensional sketch space, allowing the server to recover only the aggregate sketch for a fixed online set while protecting individual user updates.
- A Commitment-Bound Verification (CBV) method is designed to verify the aggregation state. CBV binds the participating set, aggregation weight, and aggregate sketch before fresh audit information is generated, preventing the server from modifying the result after observing the audit information.
- Security analysis demonstrates the privacy and verifiability guarantees of VCFedAdam under the sensor-network threat model. Performance evaluations show that VCFedAdam reduces computation, communication, and storage overheads with only a small loss in model accuracy, while error feedback helps compensate for the approximation error introduced by sketch compression.

The remainder of this paper is organized as follows. Section 2 reviews the related work. Section 3 introduces the technical preliminaries. In Section 4, we describe the system model, threat model, and design goals. In Section 5, we present VCFedAdam in detail. In Section 6, we provide the security analysis of VCFedAdam. Then, we evaluate the computation, communication, storage, and model utility in Section 7. Finally, we conclude the paper in Section 8.

## 2. Related Work

This section reviews recent research on privacy-preserving aggregation and VA in FL. The following subsections discuss these two research directions in detail. Table 1 provides a comprehensive comparison of representative privacy-preserving and verifiable aggregation schemes.

### 2.1. Privacy-Preserving FL

Privacy-preserving data aggregation is widely used in FL to protect local updates during collaborative training. Existing studies mainly employ HE, DP, SMC, functional encryption, and masking-based SA. These techniques allow the server to compute aggregated information without directly observing individual updates, and have been extended to resource-constrained, dynamic, and weighted aggregation settings.

A foundational line of privacy-preserving aggregation is SA. Bonawitz et al. [20] proposed a practical masking-based SA protocol that enables the server to recover only the summed update while supporting user dropouts. Following this line, Bouamama et al. [21] proposed EdgeSA to extend SA to edge computing with distributed Paillier encryption and pairing-based signatures. Fotohi et al. [22] proposed FEDANIL+, which combines CKKS-FHE, consortium blockchain, and gradient compression for privacy-preserving intelligent-enterprise FL. Yu et al. [23] proposed PrivLDFL, a functional-encryption-based scheme that supports lightweight and dynamic privacy-preserving FL through vector compression and user partitioning. He and Yu et al. [24] proposed SWAS to protect both model parameters and local data sizes in secure weighted aggregation. Ghazal et al. [25] studied generative FL for healthcare IoT data fusion with authentication and verification mechanisms. These works improve privacy protection, efficiency, and deployment flexibility; however, privacy-preserving aggregation mainly ensures that individual updates are hidden from the server, while users still need a verifiable mechanism to check whether the returned aggregate is consistent with the submitted updates and verification materials.

### 2.2. Verifiable FL

Verifiable FL incorporates verification mechanisms into the aggregation protocol to ensure the consistency, integrity, and correctness of the information used throughout the aggregation process. Xu et al. [16] proposed VerifyNet, which combines privacy-preserving aggregation with result verification by using double masking and homomorphic-hash-based proofs. Guo et al. [26] proposed VeriFL, which improves verification efficiency through linearly homomorphic hashes and commitments and reduces the communication cost of verification. Later studies further enhance VA under more practical threat models. Xie et al. [13] proposed VCFL, which considers server-user collusion and adopts optimized double masking with ECC-based commitments to support dynamic and collusion-resistant verification. Bouamama et al. [28] proposed VeSAFL, which employs a multi-aggregator architecture and combines Pedersen verifiable secret sharing with BLS signatures to authenticate local shares, partial aggregations, and final global updates.

Another line of VA introduces auxiliary or compute nodes to reduce user-side burden and assist mask recovery. Eltaras et al. [27] propose an auxiliary-node-assisted protocol, where ANs help remove masks and users verify the returned aggregate through a lightweight double-aggregation relation. Xu et al. [17] further introduce compute nodes, single-mask noise, Shamir-share-based mask reconstruction, and MAC-based checking to improve dropout tolerance and reduce user overhead. These schemes are close to the system model of VCFedAdam because auxiliary entities are used to support private aggregation and result verification. Nevertheless, existing VA schemes mainly operate on high-dimensional model updates or authenticated shares. As modern FL models may contain a large number of parameters, update submission, masking, aggregate recovery, and verification still incur substantial overhead.

Existing VA schemes mainly operate on full-dimensional model updates or authenticated shares. These schemes can be inefficient when FL models contain a large number of parameters, because update submission, masking, aggregate recovery, and verification still depend on the original model dimension. Sketch-based aggregation is a promising alternative due to its low-dimensional representation and linear aggregation property, and this work explores these advantages by combining sketch-domain verification with FedAdam-style adaptive optimization.

## 3. Preliminaries

This section introduces the basic technical tools used by VCFedAdam. We focus on three components that are repeatedly used in the protocol: key-homomorphic pseudorandom masking, domain-separated round-key derivation, and sketch-based encoding and decoding.

### 3.1. Key-Homomorphic Pseudorandom Function

VCFedAdam employs a key-homomorphic pseudorandom function (KH-PRF) to generate sketch-domain masks [29]. Let *q* be a prime modulus and let *r* be the sketch dimension, where r≪d. We define the compression ratio as ρ=r/d. We consider a function

(1) $$F^{r}:Z_{q}\times {\left\{0,1\right\}}^{*}\to Z_{q}^{r}.$$

For any two keys $k_{1},k_{2}\in Z_{q}$ and any round identifier *t*, the function satisfies

(2) $$F_{k_{1}+k_{2}}^{r}\left(t\right)=F_{k_{1}}^{r}\left(t\right)+F_{k_{2}}^{r}\left(t\right)\;\left(\mathrm{mod}\;q\right).$$

A simple realization is

(3) $$F_{k}^{r}\left(t\right)=k\cdot H_{\mathrm{RO}}\left(t\right)\;\left(\mathrm{mod}\;q\right),$$

where $H_{\mathrm{RO}}\left(t\right)\in Z_{q}^{r}$ is modeled as a random-oracle output. This linearity allows the server to remove only the aggregate mask, instead of receiving every user’s individual mask.

Specifically, $U_{i}$ and $A_{m}$ derive a sketch-mask key $a_{i,m}^{\left(t\right)}$. For a fixed online set *R*, $A_{m}$ computes the aggregate mask key

(4) $$A_{R,m}^{\left(t\right)}=\sum_{U_{i}\in R}a_{i,m}^{\left(t\right)}\;\left(\mathrm{mod}\;q\right).$$

Then the server expands $A_{R,m}^{\left(t\right)}$ into

(5) $$F_{A_{R,m}^{\left(t\right)}}^{r}\left(t\right)=\sum_{U_{i}\in R}F_{a_{i,m}^{\left(t\right)}}^{r}\left(t\right)\;\left(\mathrm{mod}\;q\right).$$

Thus, masks can be canceled at the aggregate level, while the server does not learn any individual mask key or individual sketch.

### 3.2. Domain-Separated Round-Key Derivation

During setup, each $U_{i}$ and $A_{m}$ establish a shared master seed $s_{i,m}^{\left(0\right)}$ through the key agreement algorithm KA. In each training round *t*, both parties refresh the seed by a hash-chain update:

(6) $$s_{i,m}^{\left(t\right)}=H\;\left(s_{i,m}^{\left(t-1\right)}∥t\right).$$

To avoid key reuse across protocol roles, they derive separate round keys using public domain-separation labels following NIST SP 800-108 [30]:

(7) $$a_{i,m}^{\left(t\right)}=H_{q}\;\left(s_{i,m}^{\left(t\right)}∥{\mathrm{tag}}_{\mathrm{mask}}\right),$$

(8) $$k_{i,m}^{\left(t\right)}=H_{q}\;\left(s_{i,m}^{\left(t\right)}∥{\mathrm{tag}}_{\mathrm{mac}}\right),$$

(9) $$e_{i,m}^{\left(t\right)}=H\;\left(s_{i,m}^{\left(t\right)}∥{\mathrm{tag}}_{\mathrm{enc}}\right).$$

Here, $a_{i,m}^{\left(t\right)}$ is used for sketch masking, $k_{i,m}^{\left(t\right)}$ is used for scalar tag generation and verification, and $e_{i,m}^{\left(t\right)}$ is used as the authenticated-encryption key between $U_{i}$ and $A_{m}$. The labels ${\mathrm{tag}}_{\mathrm{mask}}$, ${\mathrm{tag}}_{\mathrm{mac}}$, and ${\mathrm{tag}}_{\mathrm{enc}}$ are fixed and public. Following the principle of domain-separated key extraction and expansion [31], this design keeps the masking, verification, and encryption keys computationally separated, even though they are derived from the same evolving round seed $s_{i,m}^{\left(t\right)}$.

### 3.3. Sketch Encoding and Decoding

VCFedAdam compresses high-dimensional local updates before masking and aggregation. In round *t*, all parties derive a public sketch seed

(10) $$\sigma_{t}=H\left({\mathrm{tag}}_{\mathrm{sketch}}∥t\right),$$

and use it to generate a sketch matrix

(11) $$S_{t}\in Z_{q}^{r\times d},$$

where r≪d. For a weighted and residual-corrected local update $u_{i}^{\left(t\right)}\in Z_{q}^{d}$, user $U_{i}$ computes

(12) $$y_{i}^{\left(t\right)}=S_{t}u_{i}^{\left(t\right)}\;\left(\mathrm{mod}\;q\right),$$

where $y_{i}^{\left(t\right)}\in Z_{q}^{r}$ is the compressed sketch uploaded after masking.

The linearity of $S_{t}$ enables direct aggregation in the sketch domain:

(13) $$Y_{R}^{\left(t\right)}=\sum_{U_{i}\in R}y_{i}^{\left(t\right)}=S_{t}\sum_{U_{i}\in R}u_{i}^{\left(t\right)}\;\left(\mathrm{mod}\;q\right).$$

After the aggregate sketch passes verification, the server decodes it by

(14) $$D_{t}\left(Y_{R}^{\left(t\right)}\right)=\lambda_{s}S_{t}^{⊤}Y_{R}^{\left(t\right)},$$

where $\lambda_{s}$ is a public normalization factor determined by the sketch distribution. The decoded vector is an approximation of the full-dimensional aggregate update and is then used in the FedAdam update. Because decoding is approximate, VCFedAdam maintains a local residual at each user to feed the previous sketching error back into later rounds.

## 4. Models and Design Goal

This section describes the system model, threat model and design goals of the proposed scheme VCFedAdam.

### 4.1. System Model

VCFedAdam adopts an auxiliary-assisted aggregation architecture with three logical entities: users, ANs, and an aggregation server. In sensor-network deployments, users may be sensor-equipped edge devices, such as wearable devices, smartphones, industrial IoT terminals, vehicular units, or environmental monitoring nodes. ANs may be edge gateways, base stations, access points, roadside units, or fog nodes, while the aggregation server is implemented by an edge or cloud server.

- **User $U_{i}$.** Each $U_{i}$ holds private sensing data and performs local training when selected. Before uploading, it compresses the local update into a low-dimensional sketch, masks the sketch using pseudorandom masks derived from pairwise user-auxiliary seeds, and generates a scalar verification tag. Thus, $U_{i}$ never sends raw sensor data, plaintext model updates, or unmasked sketches to S, which limits the server’s view to protected protocol messages.
- **Auxiliary node $A_{m}$.** Each $A_{m}$ acts as a network-side helper rather than a training participant. It establishes pairwise seeds with users, derives round-specific masking, verification, and encryption keys, and distributes encrypted audit materials. After the online set (R) is fixed, it releases only aggregate recovery and verification-key materials for *R*. It does not access local datasets, plaintext updates, or individual unmasked sketches.
- **Aggregation server S.** The aggregation server S coordinates each training round by broadcasting the global model, collecting masked sketches and scalar tags, and determining the online set *R*. With auxiliary materials, it recovers only the aggregate sketch, broadcasts the aggregate result for user-side verification, and then decodes the verified sketch for the FedAdam update. Under the leakage model, S observes aggregate outputs but not individual user updates.

### 4.2. Threat Model

The following assumptions are made in our threat model. All users and ANs follow the prescribed protocol steps, but they may share their internal states and received messages with colluding parties to infer other users’ private information. The server follows the protocol during message collection and recovery, but it may attempt to infer individual sensing-model updates or return a modified aggregation result.

- **Server.** S is the main curious or misbehaving party. It receives masked sketches, aggregate recovery materials, verification messages, and final outputs. It may use these views to infer an individual user’s sensing-model update. It may also return a modified aggregate sketch during the aggregation-output stage.
- **Collusion among users.** A subset of users may collude and share their local states, protocol messages, and derived keys. Such users still follow the prescribed local computations. We allow up to |R|−2 users to collude in each round. Therefore, at least two users in the online set remain non-colluding.
- **Collusion among ANs.** A subset of auxiliary nodes may collude and share their internal states and protocol materials. We allow up to M−1 ANs to collude. Thus, at least one AN remains non-colluding in each round.
- **Cross-party collusion.** S may collude simultaneously with users and ANs. In the strongest considered case, S colludes with up to |R|−2 users and M−1 ANs. The colluding users and ANs still follow the protocol, but they may disclose their complete internal states and received protocol materials to S. At least two users and one AN remain non-colluding.

### 4.3. Design Goals

**Sensor-update privacy.** For the online set *R*, the aggregation server should obtain only the aggregate sketch required for global model updating. It should not reconstruct any individual sensing-model update, local training result, or unmasked sketch from the uploaded messages and auxiliary materials. This privacy guarantee holds when at least one AN remains non-colluding with S in each round.

**Dropout-aware aggregation.** The protocol should aggregate only those users that are available and have submitted valid masked sketches in the current round. Since sensor devices may disconnect or enter sleep states, dropped users must be excluded from both mask cancellation and verification. ANs therefore release aggregate materials only for the fixed online set (R), ensuring that unavailable users do not disturb aggregation.

**Aggregate verifiability.** Users should be able to check whether the sketch returned by S is consistent with the uploaded sketch tags. If the server modifies, replaces, or fabricates the aggregate sketch, the verification equation should fail except with negligible probability. The hidden sketch-domain challenge prevents S from constructing a forged aggregate that passes user-side verification.

**Sketch-based adaptive updating.** After the aggregate sketch is accepted, the server decodes it into an approximate global update and applies the FedAdam-based optimization rule. The protocol therefore supports adaptive FL over compressed sensing-model updates, instead of falling back to full-dimensional FedAvg aggregation. This goal preserves the efficiency of sketch-domain aggregation while maintaining compatibility with heterogeneous sensor data.

## 5. Proposed Scheme

**Figure 1:**
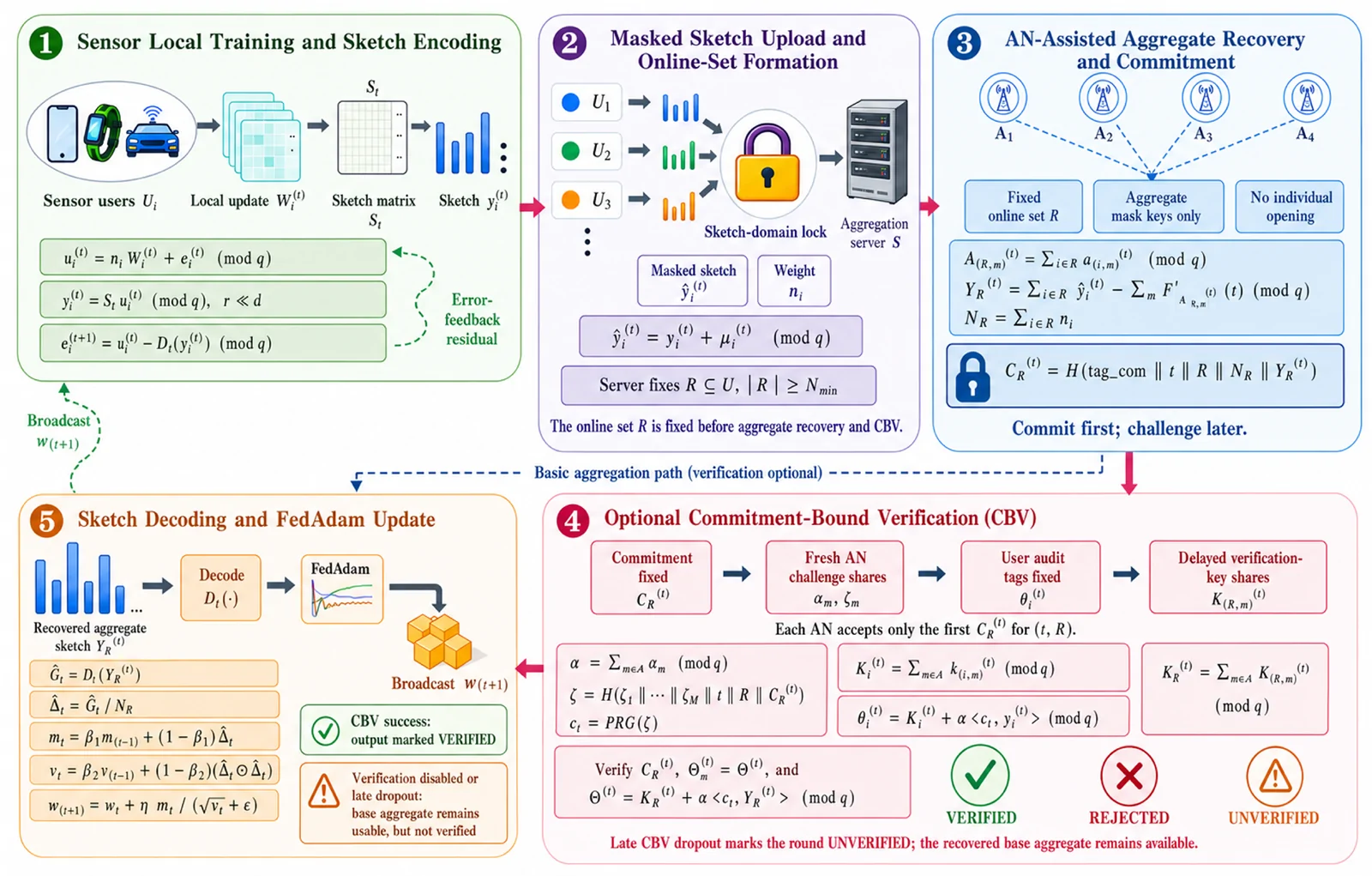
Overall workflow of VCFedAdam in DSN-FL.

**Figure 2:**
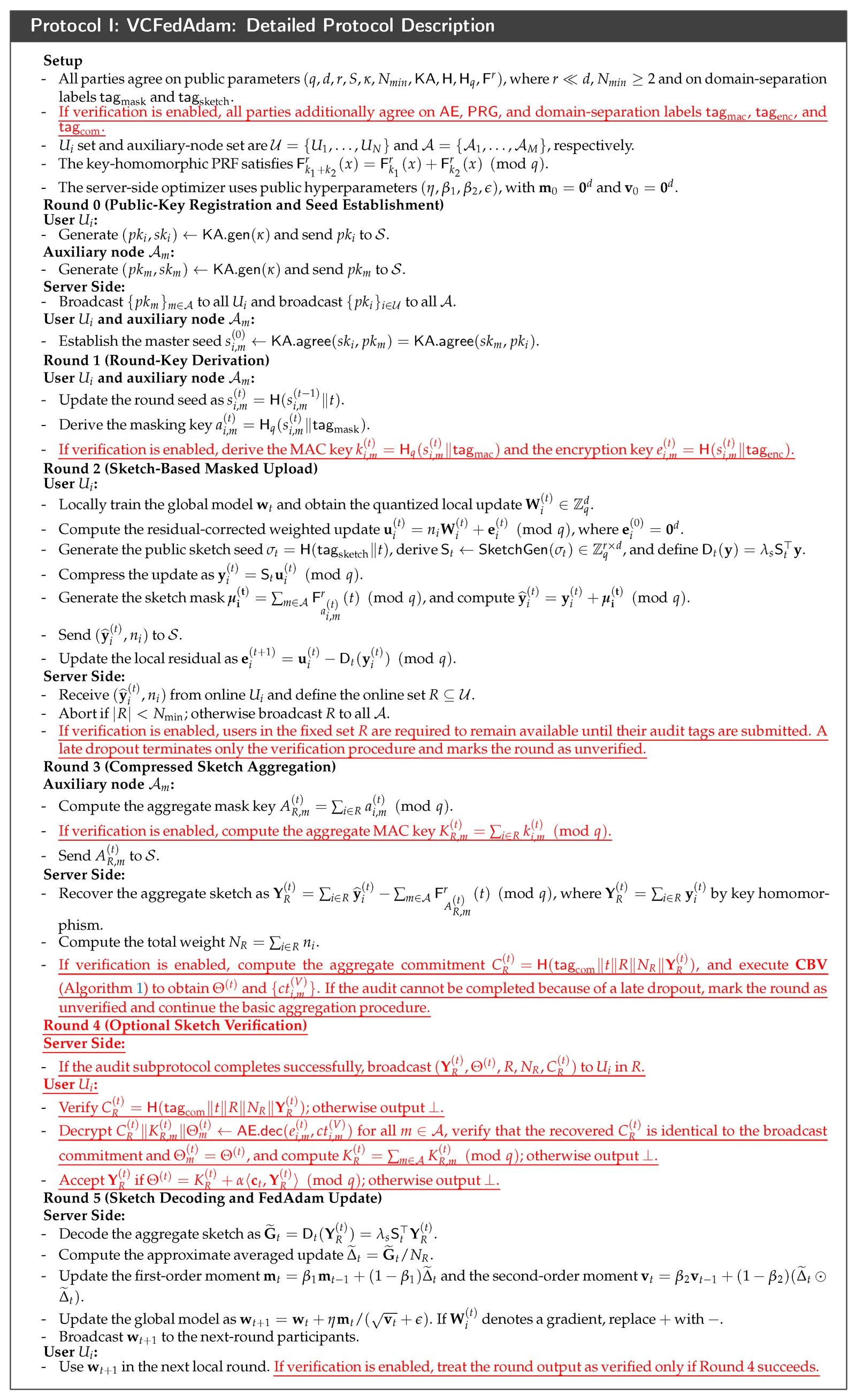
Detailed description of VCFedAdam. Black text describes the basic sketch-based secure aggregation and FedAdam update procedure. Red, underlined parts indicate the additional CBV operations, including commitment generation, audit-tag computation, and user-side aggregate verification.

This section presents VCFedAdam for privacy-preserving FL with optional verification in DSNs. The overall workflow is illustrated in Figure 1. The protocol is designed for scenarios where sensor-equipped $U_{i}$ train local sensing models but cannot afford full-dimensional SA. VCFedAdam therefore performs submission, masking, and aggregation in the sketch domain. Verification is provided as an optional extension. Each $U_{i}$ sends a masked compact sketch, while A provide only aggregate-level recovery. If verification is enabled, CBV generates the required verification materials. After the aggregate sketch is recovered, S decodes it and updates the global model using FedAdam, as specified in Figure 2. The main notations used in VCFedAdam are summarized in Appendix A.

**Algorithm 1:** Commitment-Bound Verification (CBV)**Input**: $t,R,C_{R}^{\left(t\right)},y_{i}^{\left(t\right)},k_{i,m}^{\left(t\right)},K_{R,m}^{\left(t\right)},e_{i,m}^{\left(t\right)}$, where $U_{i}\in R$, $A_{m}\in A$.**Output**: $\Theta^{\left(t\right)}$ and $ct_{i,m}^{\left(V\right)}$.**Server:**       Sends $C_{R}^{\left(t\right)}$ to all $A_{m}$.**$A_{m}$:**       Accepts only the first $C_{R}^{\left(t\right)}$ for (t,R), randomly selects $\alpha_{m},\xi_{m}\in Z_{q}$, computes $ct_{i,m}^{\left(C\right)}\leftarrow \mathrm{AE}.\mathrm{enc}(e_{i,m}^{\left(t\right)},C_{R}^{\left(t\right)}∥\alpha_{m}∥\xi_{m})$, and sends ${\left\{ct_{i,m}^{\left(C\right)}\right\}}_{i\in R}$ to the server.**Server:**       Forwards ${\left\{ct_{i,m}^{\left(C\right)}\right\}}_{m\in A}$ to each $U_{i}\in R$.**User $U_{i}$:**       Decrypts $C_{R}^{\left(t\right)}∥\alpha_{m}∥\xi_{m}\leftarrow \mathrm{AE}.\mathrm{dec}\left(e_{i,m}^{\left(t\right)},ct_{i,m}^{\left(C\right)}\right)$ for all $A_{m}$, and verifies the recovered commitments.       Computes $\alpha =\sum_{m\in A}\alpha_{m}\;\left(\mathrm{mod}\;q\right)$, $\xi =H(\xi_{1}∥\ldots ∥\xi_{M}∥t∥R∥C_{R}^{\left(t\right)})$, $c_{t}=\mathrm{PRG}\left(\xi \right)$, $K_{i}^{\left(t\right)}=\sum_{m\in A}k_{i,m}^{\left(t\right)}\;\left(\mathrm{mod}\;q\right)$, and $\theta_{i}^{\left(t\right)}=K_{i}^{\left(t\right)}+\alpha \langle c_{t},y_{i}^{\left(t\right)}\rangle \;\left(\mathrm{mod}\;q\right)$.       Computes $ct_{i,m}^{\left(\theta \right)}\leftarrow \mathrm{AE}.\mathrm{enc}\left(e_{i,m}^{\left(t\right)},C_{R}^{\left(t\right)}∥\theta_{i}^{\left(t\right)}\right)$, and sends $\left(\theta_{i}^{\left(t\right)},{\left\{ct_{i,m}^{\left(\theta \right)}\right\}}_{m\in A}\right)$ to the server.**Server:**       If any tag is missing, terminates CBV and marks the round unverified; otherwise computes $\Theta^{\left(t\right)}=\sum_{i\in R}\theta_{i}^{\left(t\right)}\;\left(\mathrm{mod}\;q\right)$ and forwards ${\left\{ct_{i,m}^{\left(\theta \right)}\right\}}_{i\in R}$ to each $A_{m}$.**$A_{m}$:**       Decrypts $C_{R}^{\left(t\right)}∥\theta_{i}^{\left(t\right)}\leftarrow \mathrm{AE}.\mathrm{dec}\left(e_{i,m}^{\left(t\right)},ct_{i,m}^{\left(\theta \right)}\right)$ for $U_{i}\in R$, verifies $C_{R}^{\left(t\right)}$, and computes $\Theta_{m}^{\left(t\right)}=\sum_{i\in R}\theta_{i}^{\left(t\right)}\;\left(\mathrm{mod}\;q\right)$.       Computes $ct_{i,m}^{\left(V\right)}\leftarrow \mathrm{AE}.\mathrm{enc}(e_{i,m}^{\left(t\right)},C_{R}^{\left(t\right)}∥K_{R,m}^{\left(t\right)}∥\Theta_{m}^{\left(t\right)})$, and sends ${\left\{ct_{i,m}^{\left(V\right)}\right\}}_{i\in R}$ to the server.**Server:**       Forwards ${\left\{ct_{i,m}^{\left(V\right)}\right\}}_{m\in A}$ to each $U_{i}\in R$.

### 5.1. Setup

In the setup phase, all parties agree on the public parameters of VCFedAdam. The sketch dimension is chosen as r≪d, which allows high-dimensional sensing-model updates to be represented by compact sketches during communication. Public domain-separation labels are assigned to masking and sketch generation, so that keys derived from the same shared seed are used for different purposes independently. If verification is enabled, additional labels are assigned to MAC generation, authenticated encryption, and commitment. AE and PRG are also used for verification. S initializes the FedAdam hyperparameters $\left(\eta ,\beta_{1},\beta_{2},\epsilon \right)$ and sets both moment vectors to zero.

### 5.2. Public-Key Registration and Seed Establishment

In this round, each $U_{i}$ and $A_{m}$ generate their key pairs locally and submit only public keys to S. S distributes these public keys but does not participate in secret generation. After receiving the corresponding public keys, $U_{i}$ and $A_{m}$ execute key agreement to obtain the common master seed $s_{i,m}^{\left(0\right)}$. This seed is later expanded into masking keys, enabling sensor U and A to maintain secure pairwise relations with limited key-management overhead. If verification is enabled, MAC and encryption keys are also derived from this seed.

### 5.3. Round Key Derivation

In round *t*, each pair $\left(U_{i},A_{m}\right)$ refreshes its shared seed through a hash-chain update and derives the key for sketch masking. If verification is enabled, MAC and encryption keys are also derived. The audit challenge is not generated in this round. It is generated later in CBV. The aggregate commitment is fixed before challenge generation.

### 5.4. Sketch Based Masked Upload

In this round, each selected $U_{i}$ trains on local sensor data and obtains a quantized local update. To account for local sample size and compression error, $U_{i}$ combines the weight $n_{i}$ with its error-feedback residual before sketching. The resulting weighted update is projected into a low-dimensional sketch using the public sketch matrix of round *t*. The sketch is then masked using auxiliary-node-derived keys. Finally, $U_{i}$ uploads the masked sketch, and weight, and updates its residual according to the decoding error. After receiving the uploads, S fixes the online set *R*. If verification is enabled, each user in *R* must remain online until its audit tag is submitted. A late dropout terminates only the verification procedure. The round is then marked as unverified. The completed basic aggregation remains valid.

### 5.5. Compressed Sketch Aggregation

After receiving uploads from available sensor U, S fixes the online set *R* and aborts if |R| is below the required threshold. Otherwise, *R* is sent to all A. By using the key-homomorphic property of $F^{r}$, A only return aggregate mask keys for *R*, rather than individual masks. S expands these aggregate keys, removes the total mask from the summed masked sketches, and obtains $Y_{R}^{\left(t\right)}$. If verification is enabled, each $A_{m}$ also computes the aggregate MAC key $K_{R,m}^{\left(t\right)}$. S computes the aggregate commitment $C_{R}^{\left(t\right)}=H({\mathrm{tag}}_{\mathrm{com}}∥t∥R∥N_{R}∥Y_{R}^{\left(t\right)})$. It then executes CBV. The audit challenge is generated after $C_{R}^{\left(t\right)}$ is fixed. If CBV is terminated by a late dropout, the recovered aggregate sketch remains available for model updating.

### 5.6. Sketch Verification

CBV generates the aggregate tag $\Theta^{\left(t\right)}$ and the verification ciphertexts. After CBV succeeds, S broadcasts the aggregate tag together with $Y_{R}^{\left(t\right)}$, the online set *R*, and the total weight. The aggregate commitment $C_{R}^{\left(t\right)}$ is also broadcast. Each online $U_{i}$ first verifies $C_{R}^{\left(t\right)}$. The user then decrypts the verification ciphertexts. Next, the user reconstructs the aggregate MAC key and verifies the returned sketch under the challenge. The recovered tag sum is also checked against $\Theta^{\left(t\right)}$. S fixes $C_{R}^{\left(t\right)}$ before the audit challenge is generated. At commitment time, the challenge is still unpredictable. Even if the challenge is disclosed later, S cannot change the committed aggregate sketch. Therefore, incorrect aggregation results are rejected except with negligible probability, while the verification overhead remains suitable for resource-constrained sensing devices.

### 5.7. Sketch Decoding and FedAdam Update

After the aggregate sketch is recovered, S decodes the aggregate sketch and normalizes the decoded result by the total online weight to obtain an approximate averaged update. This update is used to refresh the first- and second-order moments in the FedAdam optimizer, instead of being applied as a standard FedAvg update. Consequently, the cryptographic part of VCFedAdam is limited to sketch-domain recovery. Verification is optional. Adaptive optimization is performed on the decoded update. User-side error feedback further reduces the utility loss introduced by sketch compression. If Round 4 succeeds, the round output is marked as verified.

**Notes:** In VCFedAdam, the online set *R* must be fixed before mask recovery The same *R* must be used for $A_{R,m}^{\left(t\right)}$ and model updating. If verification is enabled, this *R* is also used for $K_{R,m}^{\left(t\right)}$ and CBV. Each AN releases $A_{R,m}^{\left(t\right)}$ at most once in round *t*, preventing S from obtaining aggregate openings for different user subsets. If verification is enabled, each AN accepts only the first $C_{R}^{\left(t\right)}$ for the fixed pair (t,R). If verification is enabled, the challenge $\left(\alpha ,c_{t}\right)$ is freshly generated in CBV. It is generated after $C_{R}^{\left(t\right)}$ is fixed. At commitment time, the challenge is unpredictable to S. The challenge does not need to remain secret after the commitment is fixed. Users update their residuals independently of verification. If verification is enabled, the round is marked as verified only if Round 4 succeeds. A late dropout during CBV makes the round unverified. It does not invalidate the recovered aggregate sketch. The parameters *r*, *M*, and $N_{\min}$ respectively determine the sketch compression level, the auxiliary-trust distribution, and the minimum valid online population.

## 6. Security Analysis

This section analyzes VCFedAdam under the threat model defined above. We consider the server, colluding users, colluding ANs, and cross-party collusion. Users and ANs follow the prescribed protocol but may disclose their internal states to colluding parties. The server may also return an incorrect aggregation result.

For each $U_{i}\in R$, define

$$\mu_{i,m}^{\left(t\right)}=F_{a_{i,m}^{\left(t\right)}}^{r}\left(t\right).$$

The uploaded sketch is

(15) $${\hat{y}}_{i}^{\left(t\right)}=y_{i}^{\left(t\right)}+\sum_{m\in A}\mu_{i,m}^{\left(t\right)}\;\left(\mathrm{mod}\;q\right).$$

For a fixed online set *R*, each $A_{m}$ releases

(16) $$A_{R,m}^{\left(t\right)}=\sum_{i\in R}a_{i,m}^{\left(t\right)}\;\left(\mathrm{mod}\;q\right).$$

By key homomorphism,

(17) $$F_{A_{R,m}^{\left(t\right)}}^{r}\left(t\right)=\sum_{i\in R}\mu_{i,m}^{\left(t\right)}.$$

Hence, the server recovers only

(18) $$Y_{R}^{\left(t\right)}=\sum_{i\in R}y_{i}^{\left(t\right)}.$$

### 6.1. Security Against the Server

**Theorem 1.**  *If at least one AN does not collude with the server in round t, the server cannot recover an individual sketch $y_{i}^{\left(t\right)}$ from its protocol view.*

**Proof.**  Let $A_{h}$ be a non-colluding AN. Define

(19) $$\mu_{i,h}^{\left(t\right)}=F_{a_{i,h}^{\left(t\right)}}^{r}\left(t\right).$$

After removing all mask components known to the server, its effective view of $U_{i}$ is

(20) $$z_{i}^{\left(t\right)}=y_{i}^{\left(t\right)}+\mu_{i,h}^{\left(t\right)}.$$

Since $a_{i,h}^{\left(t\right)}$ is unknown to the server, KH-PRF security gives

(21) $$\mu_{i,h}^{\left(t\right)}\approx_{c}\zeta_{i},\;\zeta_{i}\overset{\$}{\leftarrow }Z_{q}^{r}.$$

Thus, the server cannot separate $y_{i}^{\left(t\right)}$ from $z_{i}^{\left(t\right)}$.

The non-colluding AN releases only

(22) $$A_{R,h}^{\left(t\right)}=\sum_{i\in R}a_{i,h}^{\left(t\right)}\;\left(\mathrm{mod}\;q\right),$$

which reveals the aggregate mask

(23) $$F_{A_{R,h}^{\left(t\right)}}^{r}\left(t\right)=\sum_{i\in R}\mu_{i,h}^{\left(t\right)}.$$

Therefore, the server can remove the total mask but cannot recover any individual mask.

To see this more directly, consider another set of sketches ${\left\{y_{i}^{\prime (t)}\right\}}_{i\in R}$ satisfying

(24) $$\sum_{i\in R}y_{i}^{\prime (t)}=\sum_{i\in R}y_{i}^{\left(t\right)}.$$

Define

(25) $$\mu_{i,h}^{\prime (t)}=\mu_{i,h}^{\left(t\right)}+y_{i}^{\left(t\right)}-y_{i}^{\prime (t)}.$$

Then

(26) $$\sum_{i\in R}\mu_{i,h}^{\prime (t)}=\sum_{i\in R}\mu_{i,h}^{\left(t\right)}$$

and

(27) $$y_{i}^{\prime (t)}+\mu_{i,h}^{\prime (t)}=y_{i}^{\left(t\right)}+\mu_{i,h}^{\left(t\right)}.$$

Hence, both sets produce the same server view. The server can determine only the aggregate in (18), not an individual sketch. Therefore,

(28) $${\mathrm{Adv}}_{S}^{\mathrm{priv}}\leq {\mathrm{Adv}}_{\mathrm{KHPRF}}+\mathrm{negl}\left(\kappa \right).$$

□

### 6.2. Security Against Colluding Users

**Theorem 2.** 
*VCFedAdam preserves individual-sketch privacy against the collusion of up to |R|−2 protocol-following users.*

**Proof.**  Let

$$C_{U}\subset R,\;|C_{U}|\leq |R|-2$$

be the set of colluding users, and let

$$H_{U}=R\setminus C_{U}.$$

By assumption,

$$|H_{U}|\geq 2.$$

The colluding users know their own sketches. They can therefore remove their contributions from the aggregate and obtain

(29) $$Y_{H}^{\left(t\right)}=Y_{R}^{\left(t\right)}-\sum_{i\in C_{U}}y_{i}^{\left(t\right)}=\sum_{i\in H_{U}}y_{i}^{\left(t\right)}.$$

Consider the strongest case with only two non-colluding users, $U_{a}$ and $U_{b}$. Then

(30) $$Y_{H}^{\left(t\right)}=y_{a}^{\left(t\right)}+y_{b}^{\left(t\right)}.$$

For any $\delta \in Z_{q}^{r}$, define

(31) $$y_{a}^{\prime (t)}=y_{a}^{\left(t\right)}+\delta ,\;y_{b}^{\prime (t)}=y_{b}^{\left(t\right)}-\delta .$$

Then

(32) $$y_{a}^{\prime (t)}+y_{b}^{\prime (t)}=y_{a}^{\left(t\right)}+y_{b}^{\left(t\right)}=Y_{H}^{\left(t\right)}.$$

Thus, the same aggregate is consistent with many different pairs of individual sketches. Neither $y_{a}^{\left(t\right)}$ nor $y_{b}^{\left(t\right)}$ can be uniquely determined.

If only one non-colluding user $U_{h}$ remained, then

(33) $$y_{h}^{\left(t\right)}=Y_{R}^{\left(t\right)}-\sum_{i\in C_{U}}y_{i}^{\left(t\right)},$$

which would reveal its sketch directly. Therefore, the bound

$$|C_{U}|\leq |R|-2$$

is necessary.

When CBV is enabled, colluding users may disclose the challenge after receiving it. This does not enable adaptive modification because $C_{R}^{\left(t\right)}$ has already fixed the aggregate sketch. □

### 6.3. Security Against Colluding ANs

**Theorem 3.** 
*VCFedAdam preserves individual-sketch privacy against the collusion of up to M−1 ANs. When CBV is enabled, one non-colluding AN is also sufficient to keep the audit challenge unpredictable before commitment.*

**Proof.**  Let

$$C_{A}\subset A,\;\left|C_{A}\right|\leq M-1.$$

Then there exists at least one non-colluding AN

$$A_{h}\in A\setminus C_{A}.$$

For $U_{i}$, the mask can be written as

(34) $$R_{i}^{\left(t\right)}=\sum_{m\in C_{A}}F_{a_{i,m}^{\left(t\right)}}^{r}\left(t\right)+F_{a_{i,h}^{\left(t\right)}}^{r}\left(t\right).$$

After removing the components known to the colluding ANs,

(35) $$z_{i}^{\left(t\right)}=y_{i}^{\left(t\right)}+\mu_{i,h}^{\left(t\right)}.$$

Since $a_{i,h}^{\left(t\right)}$ is unknown to the colluding ANs,

(36) $$\mu_{i,h}^{\left(t\right)}\approx_{c}\zeta_{i},\;\zeta_{i}\overset{\$}{\leftarrow }Z_{q}^{r}.$$

Thus, the remaining mask hides $y_{i}^{\left(t\right)}$.

When CBV is enabled, the audit tag is

(37) $$\theta_{i}^{\left(t\right)}=K_{i}^{\left(t\right)}+\alpha \langle c_{t},y_{i}^{\left(t\right)}\rangle \;\left(\mathrm{mod}\;q\right),$$

where

(38) $$K_{i}^{\left(t\right)}=\sum_{m\in C_{A}}k_{i,m}^{\left(t\right)}+k_{i,h}^{\left(t\right)}.$$

The colluding ANs can remove their known MAC components. The remaining value is

(39) $${\tilde{\theta }}_{i}^{\left(t\right)}=k_{i,h}^{\left(t\right)}+\alpha \langle c_{t},y_{i}^{\left(t\right)}\rangle \;\left(\mathrm{mod}\;q\right).$$

Since $k_{i,h}^{\left(t\right)}$ is unknown and pseudorandom,

(40) $$\mathrm{Pr}\left[{\tilde{\theta }}_{i}^{\left(t\right)}=z|y_{i}^{\left(t\right)}\right]=\frac{1}{q}$$

for any $z\in Z_{q}$ in the ideal experiment. Hence, the audit tag does not reveal the inner product $\langle c_{t},y_{i}^{\left(t\right)}\rangle$.

The scalar challenge satisfies

(41) $$\alpha =\alpha_{h}+\sum_{m\in C_{A}}\alpha_{m}\;\left(\mathrm{mod}\;q\right).$$

Since

$$\alpha_{h}\overset{\$}{\leftarrow }Z_{q},$$

we have

(42) $$\mathrm{Pr}\left[\alpha =x|{\left\{\alpha_{m}\right\}}_{m\in C_{A}}\right]=\frac{1}{q}$$

for any $x\in Z_{q}$.

Similarly,

(43) $$\xi =H(\xi_{1}∥\cdots ∥\xi_{M}∥t∥R∥C_{R}^{\left(t\right)}).$$

At least one component $\xi_{h}$ is unknown when $C_{R}^{\left(t\right)}$ is fixed. Therefore,

$$c_{t}=\mathrm{PRG}\left(\xi \right)$$

remains computationally unpredictable at commitment time.

Hence, one non-colluding AN provides an unknown mask component, an unknown MAC component, and an unpredictable challenge contribution. This is sufficient to preserve privacy against up to M−1 colluding ANs and to support CBV security. □

### 6.4. Security Against Cross-Party Collusion

We finally consider the strongest adversary in our threat model. The server may collude with up to |R|−2 users and M−1 ANs. The colluding users and ANs follow the protocol, but may disclose their complete internal states to the server.

**Theorem 4.** 
*Assume that at least two users and one AN remain non-colluding. Then VCFedAdam preserves the privacy of each non-colluding user’s sketch under cross-party collusion. If CBV is enabled, an incorrect aggregate is accepted with probability at most*

$$\frac{1}{q}+\mathrm{negl}\left(\kappa \right).$$

**Proof.**  Let

$$C_{U}\subset R,\;\left|C_{U}\right|\leq N-2$$

be the colluding users, and let

$$C_{A}\subset A,\;\left|C_{A}\right|\leq M-1$$

be the colluding ANs.

We consider the strongest case. Let $U_{a}$ and $U_{b}$ be the only two non-colluding users. Let $A_{h}$ be the only non-colluding AN.

*Privacy.* The server learns all mask components generated by the colluding ANs. It can therefore reduce the two masked sketches to

(44) $$z_{a}^{\left(t\right)}=y_{a}^{\left(t\right)}+\mu_{a,h}^{\left(t\right)},\;z_{b}^{\left(t\right)}=y_{b}^{\left(t\right)}+\mu_{b,h}^{\left(t\right)},$$

where

$$\mu_{a,h}^{\left(t\right)}=F_{a_{a,h}^{\left(t\right)}}^{r}\left(t\right),\;\mu_{b,h}^{\left(t\right)}=F_{a_{b,h}^{\left(t\right)}}^{r}\left(t\right).$$

The colluding users also reveal their keys shared with $A_{h}$. Hence, from the aggregate key $A_{R,h}^{\left(t\right)}$, the adversary can derive only

(45) $$a_{ab}^{\left(t\right)}=A_{R,h}^{\left(t\right)}-\sum_{i\in C_{U}}a_{i,h}^{\left(t\right)}=a_{a,h}^{\left(t\right)}+a_{b,h}^{\left(t\right)}\;\left(\mathrm{mod}\;q\right).$$

By key homomorphism,

(46) $$F_{a_{ab}^{\left(t\right)}}^{r}\left(t\right)=\mu_{a,h}^{\left(t\right)}+\mu_{b,h}^{\left(t\right)}.$$

Therefore,

(47) $$\begin{array}{cc} z_{a}^{\left(t\right)}+z_{b}^{\left(t\right)}-F_{a_{ab}^{\left(t\right)}}^{r}\left(t\right)\; & =y_{a}^{\left(t\right)}+y_{b}^{\left(t\right)}. \end{array}$$

Thus, the strongest adversary still obtains only the sum of the two non-colluding sketches.

For any

$$\delta \in Z_{q}^{r},$$

consider another candidate pair

(48) $$y_{a}^{\prime (t)}=y_{a}^{\left(t\right)}+\delta ,\;y_{b}^{\prime (t)}=y_{b}^{\left(t\right)}-\delta .$$

Clearly,

(49) $$y_{a}^{\prime (t)}+y_{b}^{\prime (t)}=y_{a}^{\left(t\right)}+y_{b}^{\left(t\right)}.$$

In the KH-PRF hybrid, define

(50) $$\mu_{a,h}^{\prime (t)}=\mu_{a,h}^{\left(t\right)}-\delta ,\;\mu_{b,h}^{\prime (t)}=\mu_{b,h}^{\left(t\right)}+\delta .$$

Then

(51) $$\mu_{a,h}^{\prime (t)}+\mu_{b,h}^{\prime (t)}=\mu_{a,h}^{\left(t\right)}+\mu_{b,h}^{\left(t\right)},$$

and

(52) $$y_{a}^{\prime (t)}+\mu_{a,h}^{\prime (t)}=z_{a}^{\left(t\right)},\;y_{b}^{\prime (t)}+\mu_{b,h}^{\prime (t)}=z_{b}^{\left(t\right)}.$$

Hence, different individual sketches are consistent with the same adversarial view. The adversary can determine $y_{a}^{\left(t\right)}+y_{b}^{\left(t\right)}$, but cannot uniquely determine either component.

Under KH-PRF security,

(53) $${\mathrm{Adv}}_{\mathrm{cross}}^{\mathrm{priv}}\leq {\mathrm{Adv}}_{\mathrm{KHPRF}}+\mathrm{negl}\left(\kappa \right).$$

*Verifiability.* When CBV is enabled, the valid aggregate tag satisfies

(54) $$\begin{array}{cc} \Theta^{\left(t\right)}\; & =\sum_{i\in R}\theta_{i}^{\left(t\right)}\\ \; & =\sum_{i\in R}\left(K_{i}^{\left(t\right)}+\alpha \langle c_{t},y_{i}^{\left(t\right)}\rangle \right)\\ \; & =K_{R}^{\left(t\right)}+\alpha \langle c_{t},Y_{R}^{\left(t\right)}\rangle \;\left(\mathrm{mod}\;q\right). \end{array}$$

Suppose the server wants to return

(55) $$Y_{R}^{*}=Y_{R}^{\left(t\right)}+\Delta ,\;\Delta \neq 0.$$

Before the challenge is generated, the server must fix

(56) $$C_{R}^{\left(t\right)}=H({\mathrm{tag}}_{\mathrm{com}}∥t∥R∥N_{R}∥Y_{R}^{*}).$$

The non-colluding AN then generates fresh $\alpha_{h}$ and $\xi_{h}$. Thus, the complete $\left(\alpha ,c_{t}\right)$ is unpredictable when Δ is fixed.

Even if the colluding users disclose the complete challenge afterward, the committed aggregate cannot be adapted to that challenge.

For $Y_{R}^{*}$ to pass the original verification equation, it must satisfy

(57) $$\Theta^{\left(t\right)}=K_{R}^{\left(t\right)}+\alpha \langle c_{t},Y_{R}^{*}\rangle \;\left(\mathrm{mod}\;q\right).$$

Combining (54), (55) and (57) gives

(58) $$\langle c_{t},\Delta \rangle =0\;\left(\mathrm{mod}\;q\right).$$

Since Δ≠0, there exists an index *j* such that $\Delta_{j}\neq 0$. For fixed ${\left\{c_{\ell }\right\}}_{\ell \neq j}$, Equation (58) uniquely determines

(59) $$c_{j}=-\Delta_{j}^{-1}\sum_{\ell \neq j}c_{\ell }\Delta_{\ell }\;\left(\mathrm{mod}\;q\right).$$

Therefore, among the $q^{r}$ possible challenge vectors, only $q^{r-1}$ satisfy the forgery condition. Hence,

(60) $$\mathrm{Pr}\left[\langle c_{t},\Delta \rangle =0\right]=\frac{q^{r-1}}{q^{r}}=\frac{1}{q}.$$

The server also cannot replace the committed aggregate after seeing the challenge without finding a collision in H. Moreover, CBV binds the aggregate tag and the aggregate MAC materials to the accepted commitment through authenticated encryption. Each AN accepts only the first $C_{R}^{\left(t\right)}$ for the fixed pair (t,R).

Thus,

(61) $${\mathrm{Adv}}_{\mathrm{cross}}^{\mathrm{ver}}\leq \frac{1}{q}+{\mathrm{Adv}}_{H}^{\mathrm{coll}}+{\mathrm{Adv}}_{\mathrm{AE}}^{\mathrm{forge}}+{\mathrm{Adv}}_{\mathrm{PRG}}+\mathrm{negl}\left(\kappa \right).$$

Therefore,

(62) $${\mathrm{Adv}}_{\mathrm{cross}}^{\mathrm{ver}}\leq \frac{1}{q}+\mathrm{negl}\left(\kappa \right).$$

VCFedAdam therefore preserves individual-sketch privacy against the strongest cross-party collusion considered in this work. CBV also prevents adaptive aggregate forgery with overwhelming probability. □

## 7. Performance Evaluation

In this section, we evaluate the efficiency and model utility of VCFedAdam. The proposed scheme is compared with EVP [27], EVSA [17], VeSAFL [28], and VCFL [13], which are representative verifiable or privacy-preserving aggregation protocols with auxiliary-assisted, verification-based, or collusion-resistant designs. Unless otherwise stated, we set the number of users to N=100, the number of ANs to M=5, and the model dimension to d=10,000. For VCFedAdam, the default compression ratio is ρ=0.25, and the sketch dimension is r=ρd=2500. All schemes use the same cryptographic parameter length. For compressed schemes, vector-related computation and communication are measured over the compressed dimension.

The prototype was implemented in Python 3.13.5 and executed on a Windows 11 machine equipped with an AMD Ryzen 9 8945HX CPU with 16 cores and 32 logical threads, and 32 GB RAM. The implementation includes the main cryptographic kernels required by the compared protocols, including Diffie–Hellman key agreement, pseudorandom expansion, modular masking, hash/MAC generation, Shamir-style recovery, modular exponentiation, pairing-related operations when required, aggregation, verification, and sketch decoding.

### 7.1. Computation Overhead

We first analyze the computation complexity of different schemes. Let $C_{\mathrm{DH}}$, $C_{\exp}$, and $C_{\mathrm{pair}}$ denote the costs of one Diffie–Hellman key agreement, one modular exponentiation, and one pairing-related operation, respectively. Here, |R| denote the number of online users in one round. As shown in Table 2, full-dimensional masking schemes incur costs proportional to *d*, while VCFedAdam moves masking, recovery, and verification into the sketch domain. Therefore, the server-side cost of VCFedAdam is reduced to O(|R|r+d), where r≪d.

The phase-level computation overhead under different dropout rates is further summarized in Table 3. EVP and EVSA do not include a separate verification phase in this measurement, while VeSAFL, VCFL, and VCFedAdam include the verification-related cost. For N=100 and no dropout, VCFedAdam requires 53.21 ms for key sharing, 73.70 ms for masked upload, 1.63 ms for unmasking and aggregation, and 5.25 ms for verification. The verification cost remains lightweight because VCFedAdam verifies only a scalar sketch tag rather than a full-dimensional authenticated vector.

**Figure 3:**
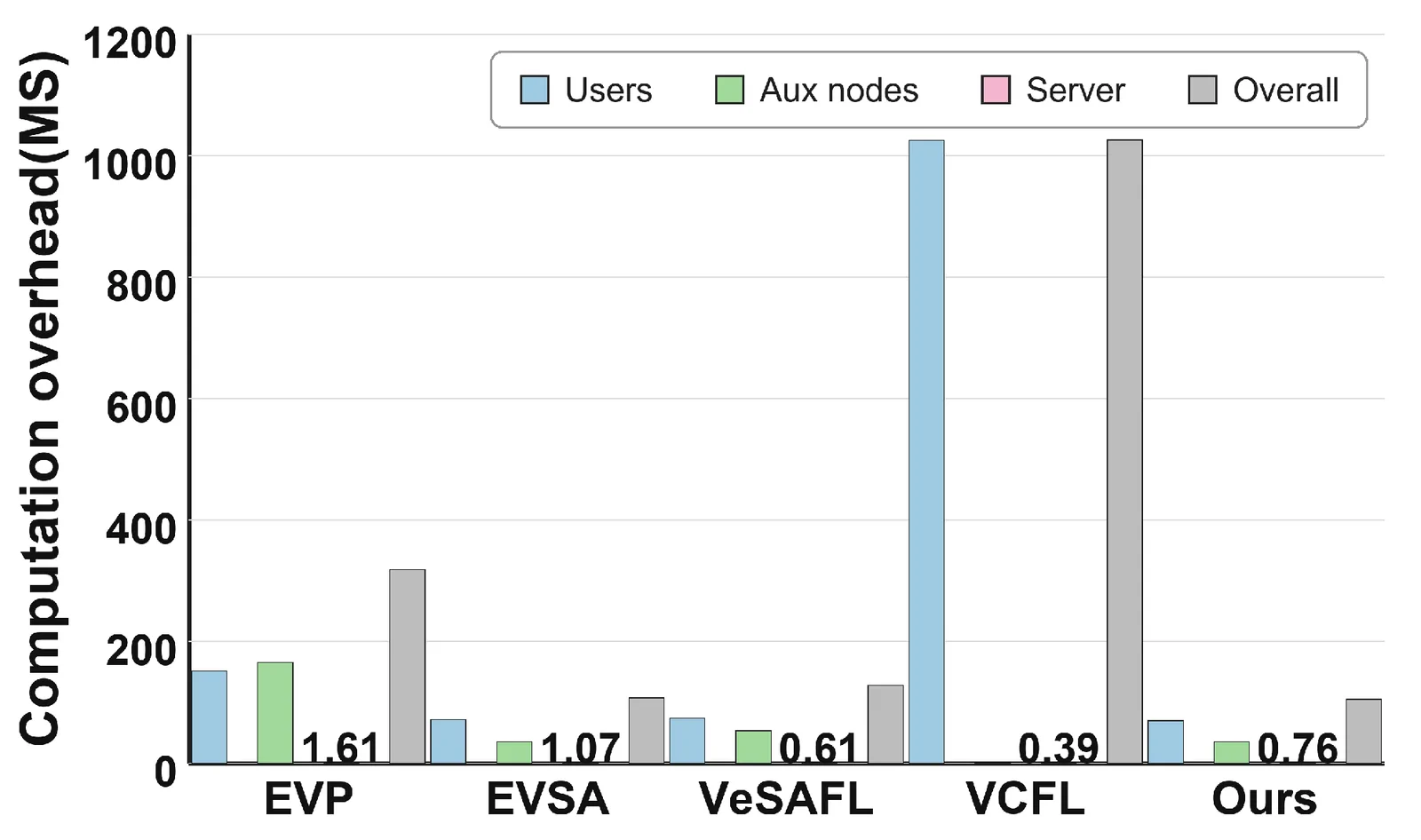
Computation overhead breakdown under N=100 and M=5.

The computation breakdown for N=100 and M=5 is shown in Figure 3. VCFL has the largest overall computation overhead because its masking and dropout-recovery operations scale poorly with the user population. EVP also incurs a considerable cost since both users and ANs perform full-dimensional masking. In contrast, VCFedAdam keeps the server cost below 1 ms in this setting and maintains an overall overhead close to EVSA, while additionally supporting sketch-domain verification and FedAdam-style adaptive updating.

**Figure 4:**
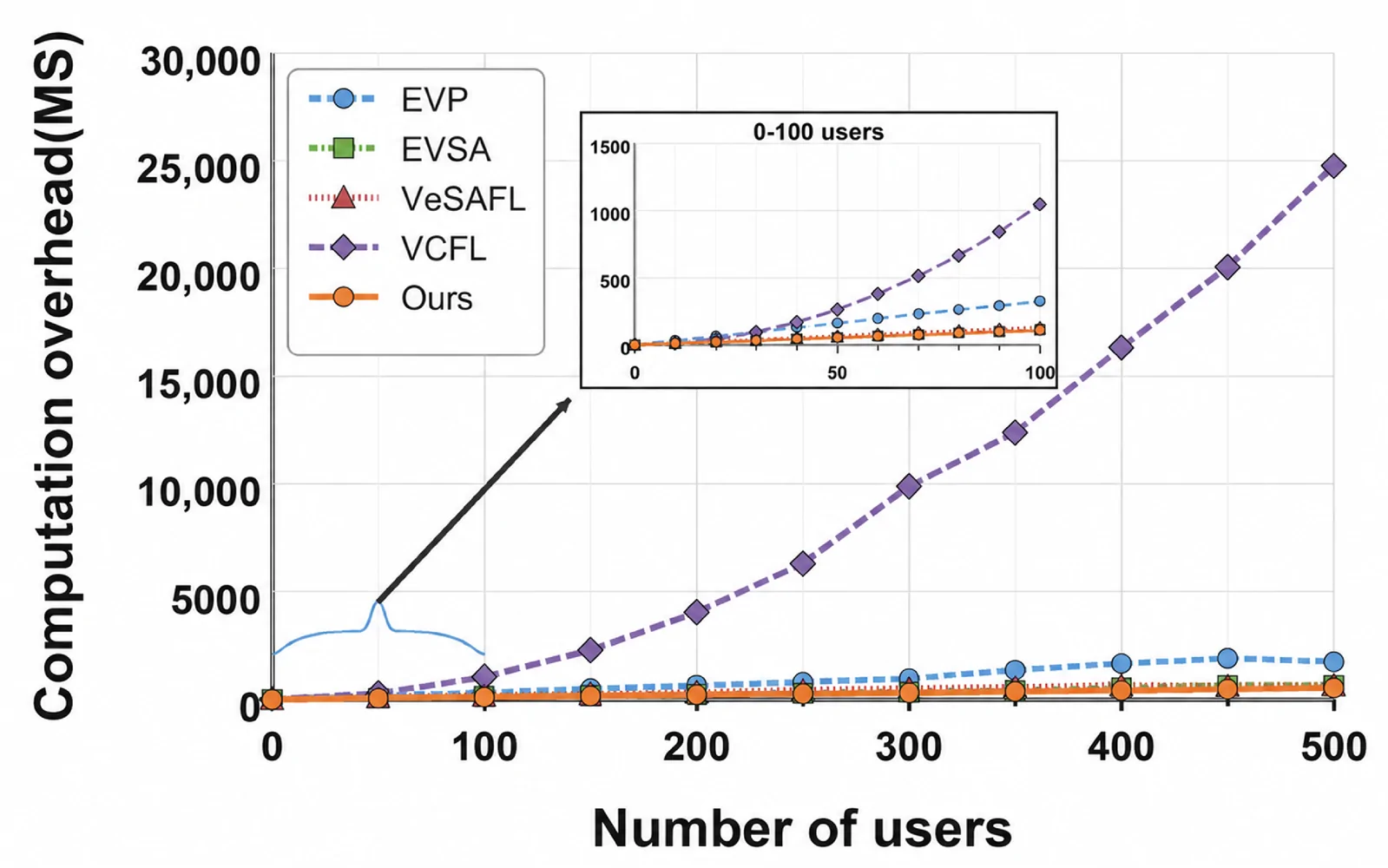
Total computation overhead with different numbers of users.

The total computation overhead under an increasing number of users from 0 to 500 is shown in Figure 4. The overhead of VCFL grows rapidly as the number of users increases, mainly due to pairwise masking and dropout-recovery operations. EVP grows more moderately but still requires full-dimensional computation. VCFedAdam scales more gently because the dominant aggregation and verification operations are performed over the sketch dimension *r* rather than the original dimension *d*.

### 7.2. Communication Overhead

**Figure 5:**
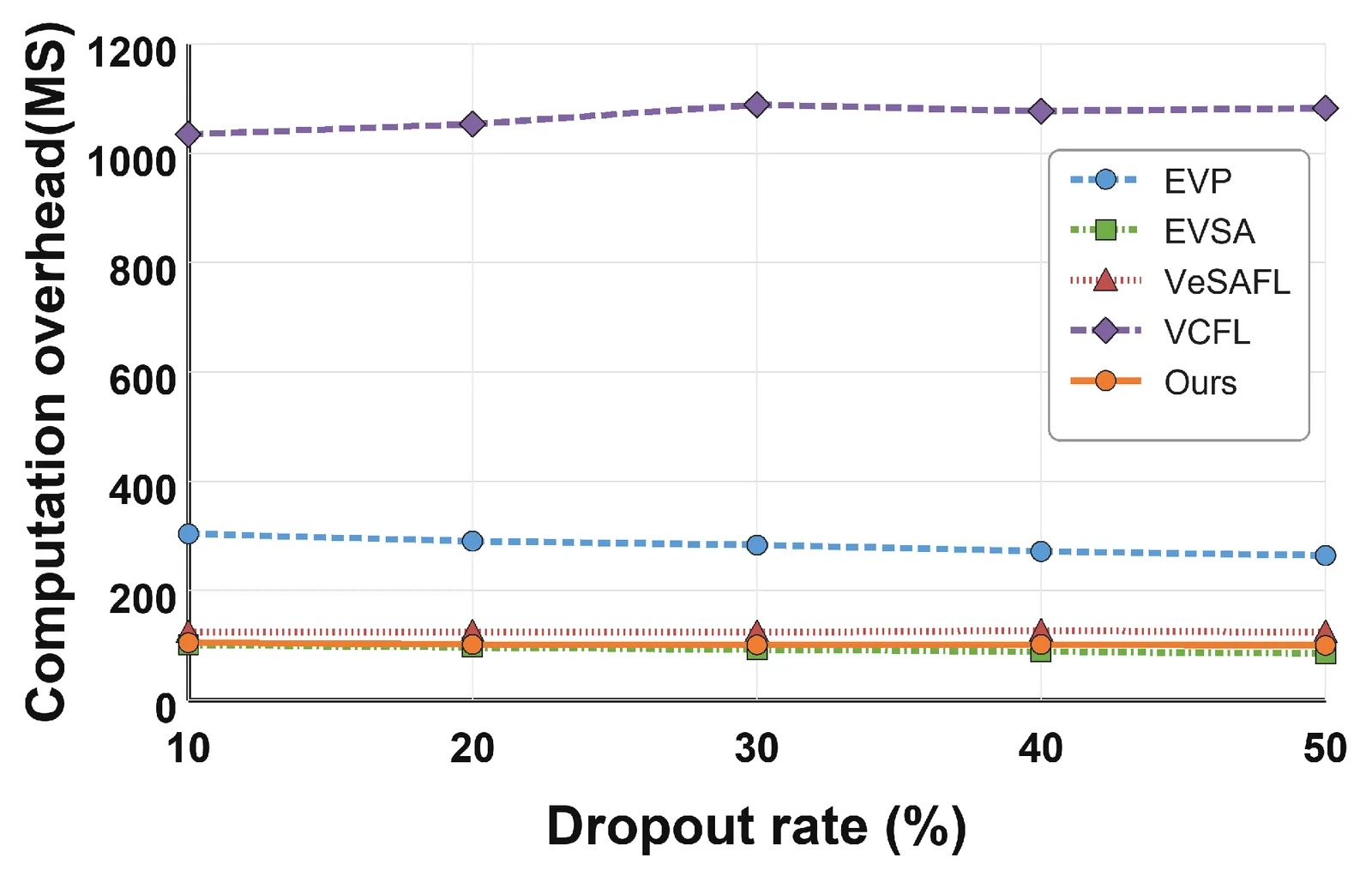
Computation overhead under different dropout rates.

The computation overhead under dropout rates from 10% to 50% is evaluated in Figure 5. The computation overhead of VCFedAdam remains stable under increasing dropout rates because ANs release aggregate recovery materials only for the fixed online set. This avoids repeated reconstruction of individual masks and prevents dropped users from introducing additional unmasking operations.

We next compare the communication complexity. Let κ denote the bit length of cryptographic seeds, keys, ciphertext components, or scalar authentication materials. As shown in Table 4, the total communication complexity of VCFedAdam is O(|R|r+NMκ). Compared with full-dimensional schemes, the vector transmission cost is reduced from the original model dimension *d* to the sketch dimension *r*. Compared with schemes dominated by pairwise or multi-aggregator communication, VCFedAdam avoids quadratic communication growth in the user dimension.

**Figure 6:**
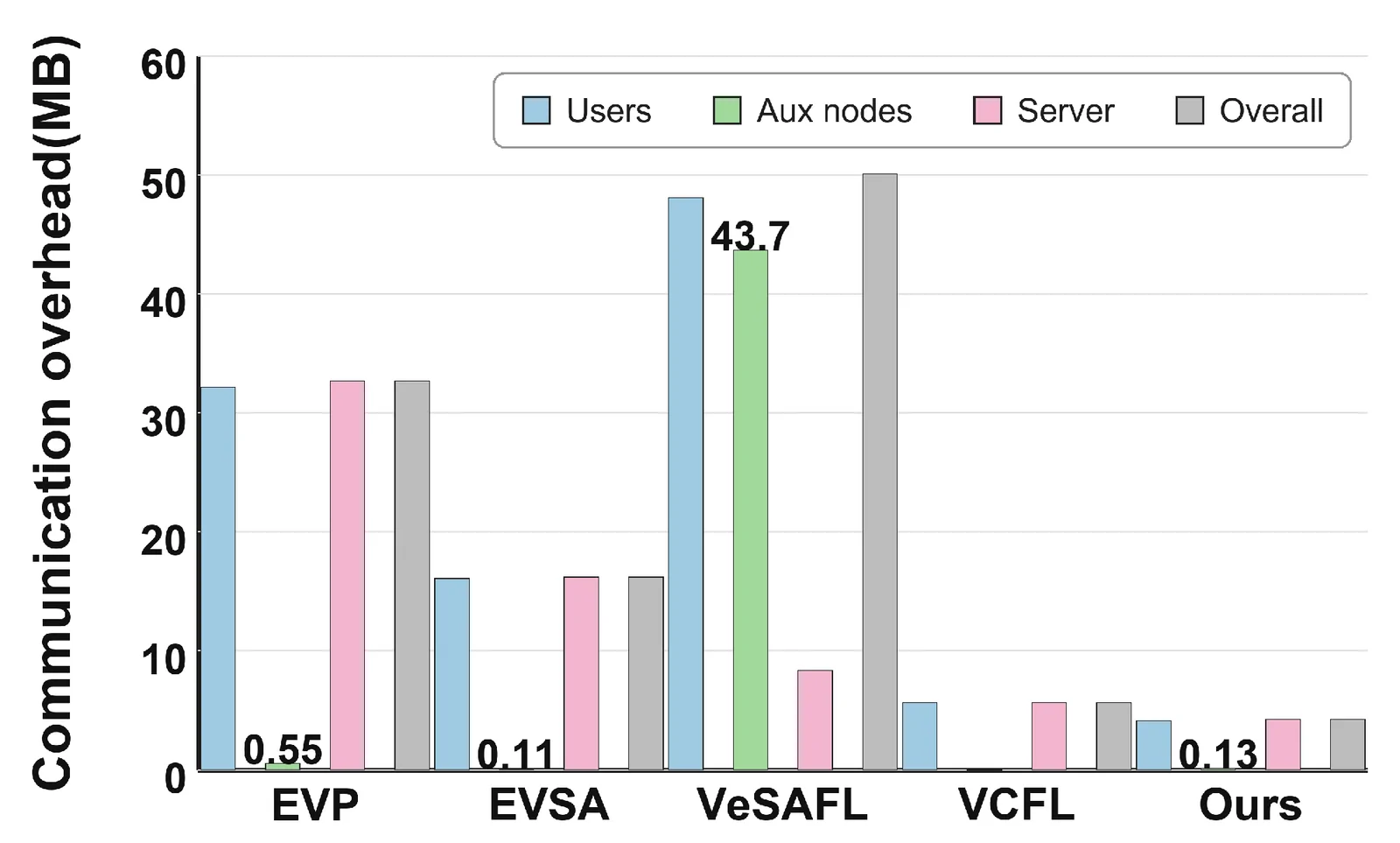
Communication overhead breakdown under N=100 and M=5.

The communication breakdown under N=100 and M=5 is shown in Figure 6. All schemes involve server-side communication because the server collects uploads, coordinates the online set, forwards auxiliary materials, and broadcasts aggregation outputs. VeSAFL shows the largest user and auxiliary-node communication overhead due to multi-aggregator and authenticated-share transmission. EVP and EVSA also transmit full-dimensional masked updates. VCFedAdam reduces overall communication by sending compressed sketches and scalar verification tags instead of full-dimensional authenticated vectors.

**Figure 7:**
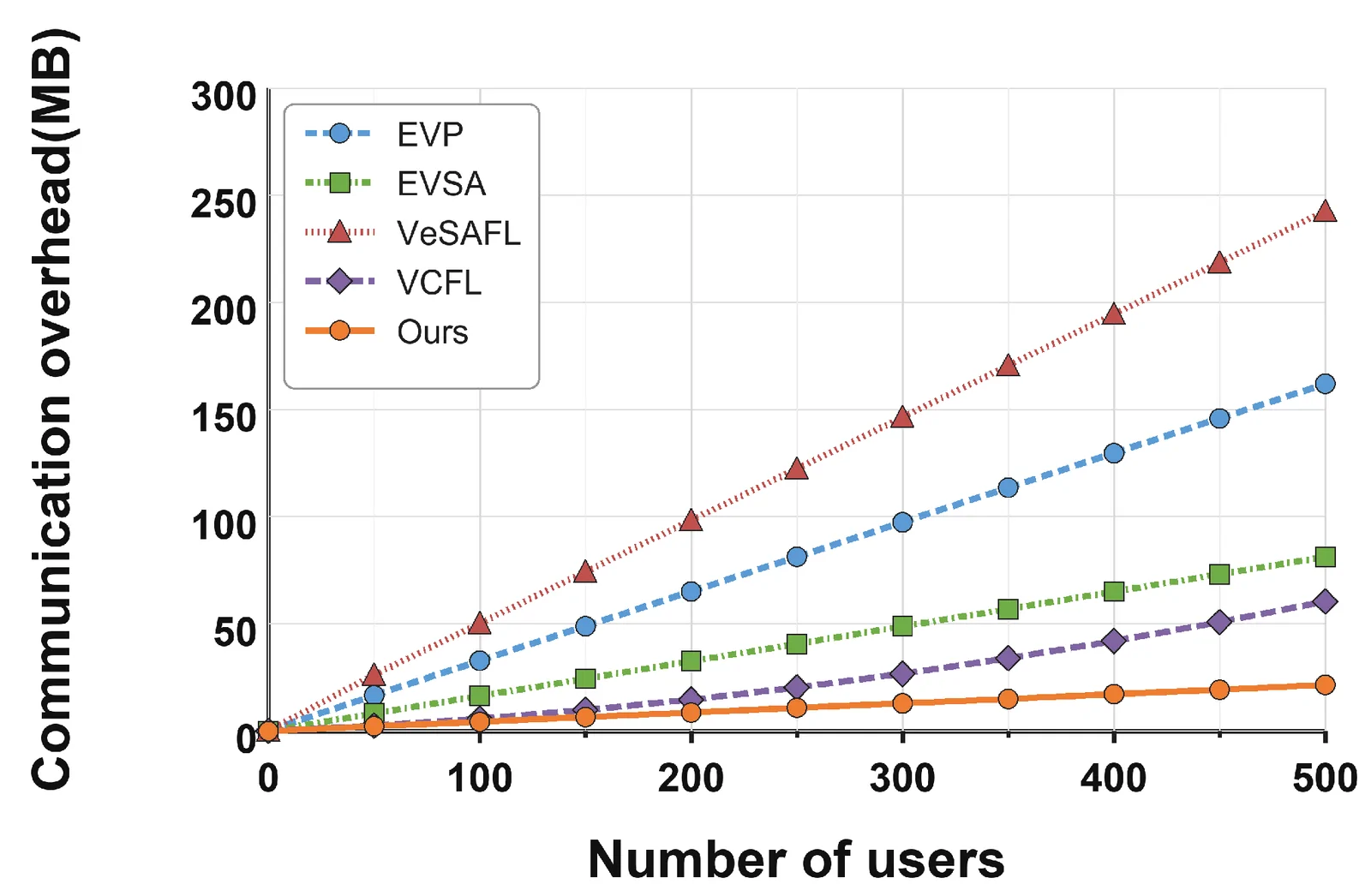
Total communication overhead with different numbers of users.

The total communication overhead under an increasing user population from 0 to 500 is reported in Figure 7. The communication overhead of VeSAFL and EVP grows quickly because their transmitted payloads remain tied to full-dimensional vectors or multi-party authentication materials. In contrast, VCFedAdam grows slowly with the number of users, since each online user uploads only a sketch-domain vector and a small scalar tag.

**Figure 8:**
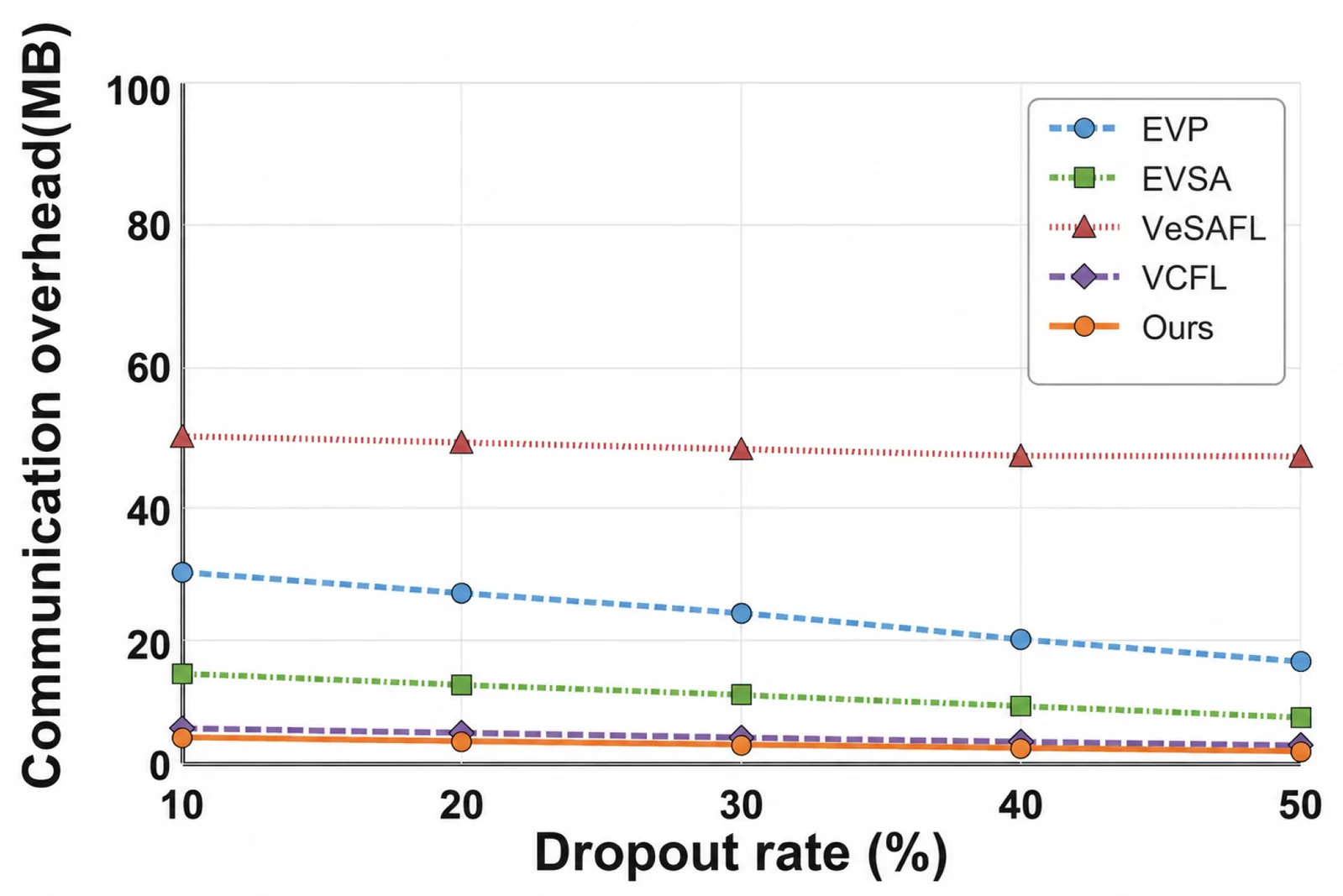
Communication overhead under different dropout rates.

The dropout experiment in Figure 8 shows that VCFedAdam maintains the lowest communication overhead across all dropout rates. As the dropout rate increases, fewer online users contribute sketch uploads, and the transmitted aggregate materials shrink accordingly. Since recovery and verification are bound to the fixed online set, no additional communication is required to reconstruct dropped-user masks.

**Figure 9:**
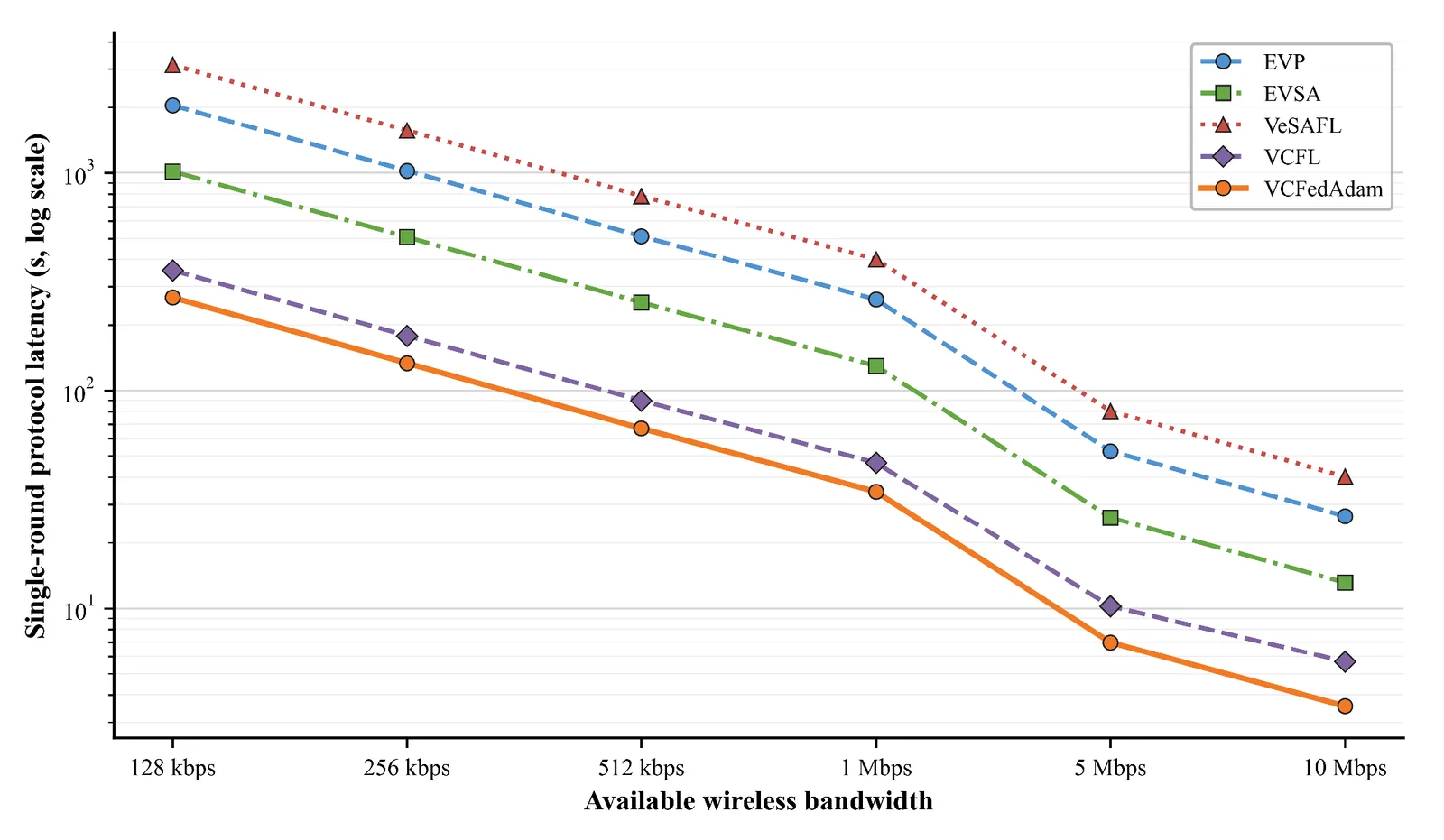
Single-round protocol latency under different wireless bandwidths.

The single-round protocol latency under varying wireless bandwidths is shown in Figure 9. The latency of all schemes decreases as the available bandwidth increases. VCFedAdam achieves the lowest latency at all tested bandwidths. Its advantage is especially clear under bandwidth-constrained settings such as 128 kbps and 256 kbps. At 128 kbps, the latency of VCFedAdam is about 270 s, while EVP, EVSA, VeSAFL, and VCFL require much longer times. When the bandwidth increases to 10 Mbps, the latency of VCFedAdam decreases to about 3.5 s and remains lower than the other schemes. These results show that the reduced communication volume of VCFedAdam can effectively shorten the training round, especially in low-bandwidth edge networks.

As shown in Table 5, sketch compression gives the main efficiency gain. It reduces computation by 17.79% and communication by 73.05% compared with VCFedAdam-FD. Residual correction adds almost no extra overhead. CBV increases computation by 20.33% and communication by 113.50% because it requires extra verification messages. Even so, the complete VCFedAdam still uses much less computation and communication than traditional SA, with reductions of 90.25% and 75.38%, respectively. Increasing *M* from 1 to 5 mainly increases computation, while the change in communication is small. The last three rows further show the effect of the compression ratio. As ρ decreases from 0.25 to 0.10 and 0.05, the computation overhead decreases from 133.79 ms to 127.94 ms and 122.37 ms, respectively. The communication overhead is further reduced from 4.428 MB to 2.024 MB and 1.217 MB. This shows that a lower compression ratio mainly provides a larger reduction in communication overhead. Therefore, a smaller ρ is more suitable for bandwidth-constrained edge networks. A larger ρ can be selected when sufficient bandwidth is available and higher model accuracy is preferred.

### 7.3. Storage Overhead

The storage overhead introduced by VCFedAdam is reported in Table 6. A user stores the pairwise seeds associated with ANs and the local residual vector used by error feedback. Single AN stores user-related seeds and round scalars. The server stores public keys, the aggregate sketch, and the FedAdam moment states. Under N=100, M=5, d=10,000, and ρ=0.25, the concrete storage overhead is 80.16 KB for each user, 3.33 KB for each AN, 266.72 KB for the server, and 8.30 MB in total. This storage cost is acceptable for sensor-network settings because the dominant user-side additional state is the residual vector, which is necessary for compensating sketch-decoding error.

### 7.4. Accuracy

Finally, we evaluate whether sketch compression affects model utility. The accuracy experiments are not intended to pursue state-of-the-art image classification performance; instead, they isolate the utility loss introduced by sketch compression and assess the benefit of error feedback. For both tasks, we use a linear softmax classifier consisting of a single fully connected layer that maps the input features to ten class logits and is trained using cross-entropy loss. FedAdam [32] without sketch compression serves as the baseline and is compared with VCFedAdam under different compression ratios while keeping the model architecture and training configuration unchanged.

We evaluated the model utility of VCFedAdam on two popular image-classification datasets, CIFAR-10 and MNIST:

- **CIFAR-10** [33] contains 50,000 training images and 10,000 test images from 10 object classes. Each RGB image is flattened into a 3072-dimensional vector and augmented with one bias coordinate.
- **MNIST** [34] contains 60,000 training images and 10,000 test images from 10 handwritten-digit classes. Each grayscale image is flattened into a 784-dimensional vector and augmented with one bias coordinate.

For both datasets, the global model is a linear softmax classifier trained using cross-entropy loss. The training data are distributed among 50 users under both IID and non-IID settings. In the IID setting, samples are randomly and approximately evenly assigned to users. In the non-IID setting, samples are sorted by class and divided into 100 label-skewed shards, with each user randomly receiving two shards. In each communication round, 10 users are randomly selected to perform local stochastic-gradient updates, while the server updates the global model using FedAdam. Unless otherwise stated, VCFedAdam uses a compression ratio of ρ=0.25 with error feedback enabled. The same model architecture and training configuration are used in both settings to ensure a fair comparison.

**Figure 10:**
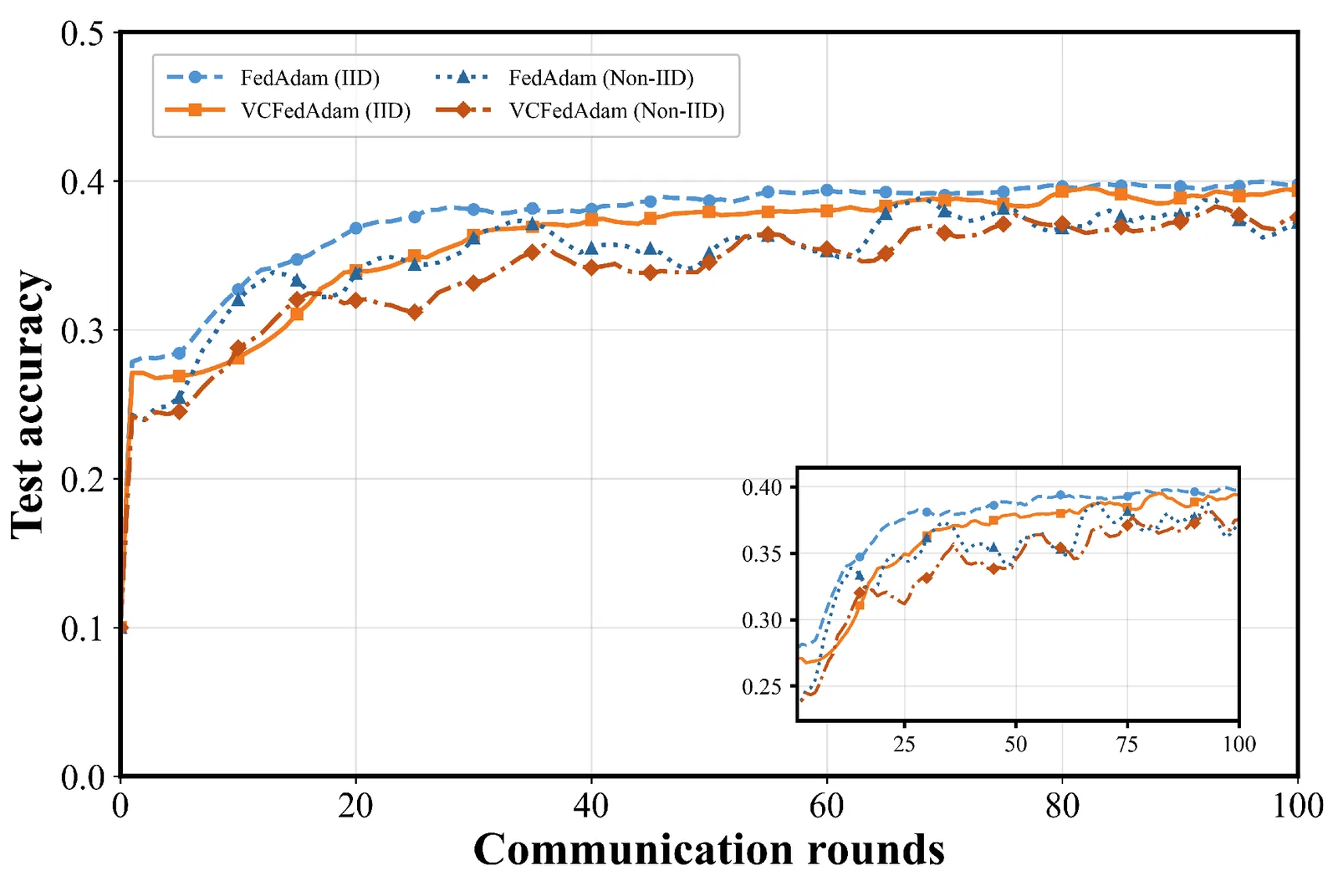
CIFAR-10 test accuracy over communication rounds under IID and Non-IID data distributions.

The CIFAR-10 test accuracy over 100 communication rounds is presented in Figure 10. Under the IID setting, final accuracies of 39.71% and 39.17% are achieved by FedAdam and VCFedAdam, respectively, resulting in a gap of only 0.54 percentage points. Under the non-IID setting, larger fluctuations are observed because of the label-skewed data distribution. Nevertheless, a convergence trajectory comparable to that of FedAdam is maintained by VCFedAdam, indicating that sketch compression remains effective under heterogeneous client data.

**Figure 11:**
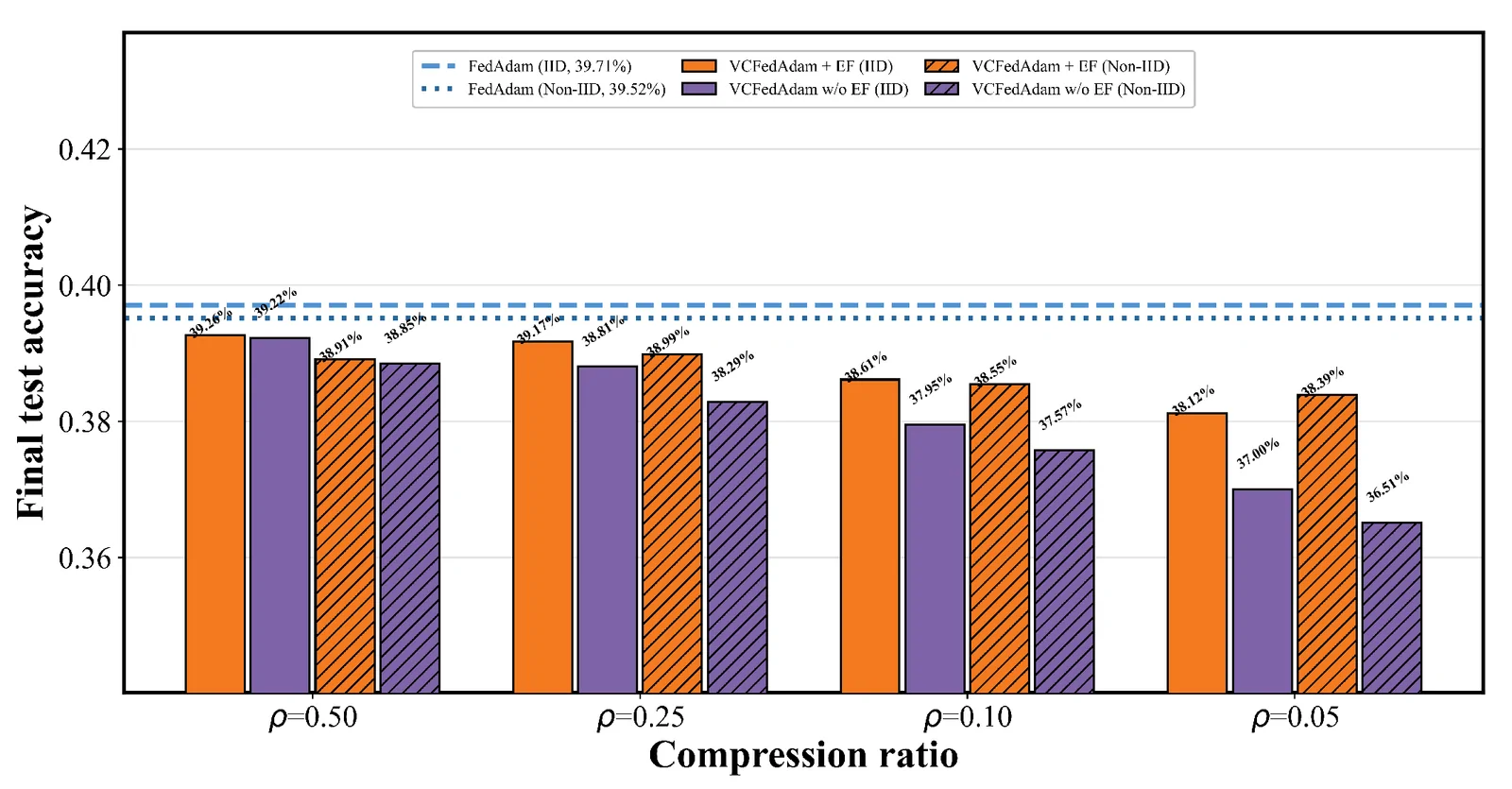
CIFAR-10 accuracy under different compression ratios and data distributions.

The final CIFAR-10 accuracy under different compression ratios is reported in Figure 11. Under both IID and non-IID settings, accuracy close to the corresponding FedAdam baseline is retained when error feedback is enabled. A gradual reduction is observed as the compression ratio decreases. Without error feedback, a larger accuracy loss is introduced, particularly at ρ=0.10 and ρ=0.05. These results confirm that accumulated sketch-decoding errors are effectively compensated for by error feedback.

**Figure 12:**
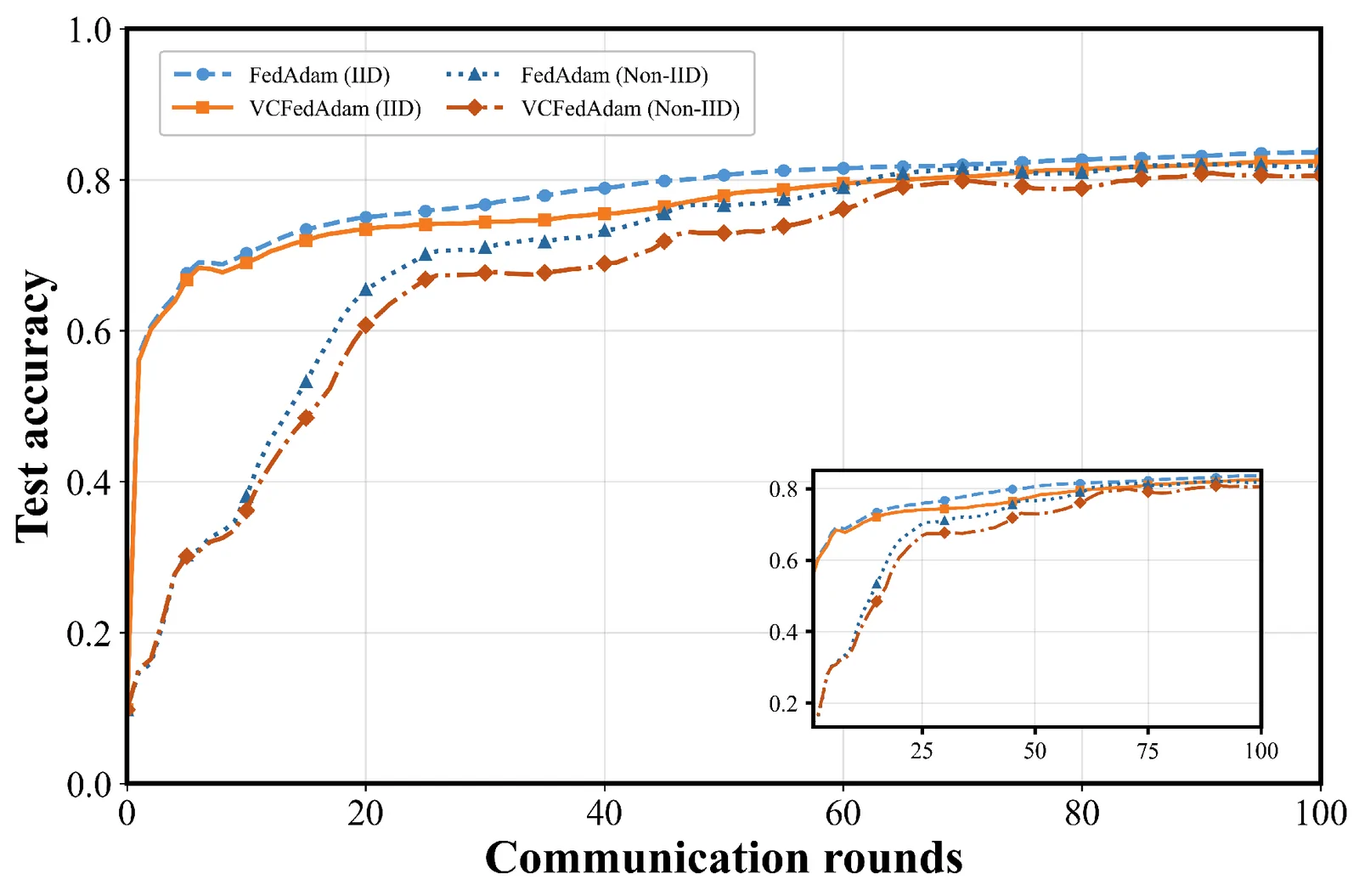
MNIST test accuracy over communication rounds under IID and Non-IID data distributions.

The MNIST convergence results under IID and non-IID data distributions are illustrated in Figure 12. Under the IID setting, final accuracies of 83.58% and 82.42% are obtained by FedAdam and VCFedAdam, respectively. Under the non-IID setting, slower early-stage convergence and greater fluctuations are observed. However, comparable final accuracy is eventually achieved, demonstrating the robustness of VCFedAdam to statistical heterogeneity.

**Figure 13:**
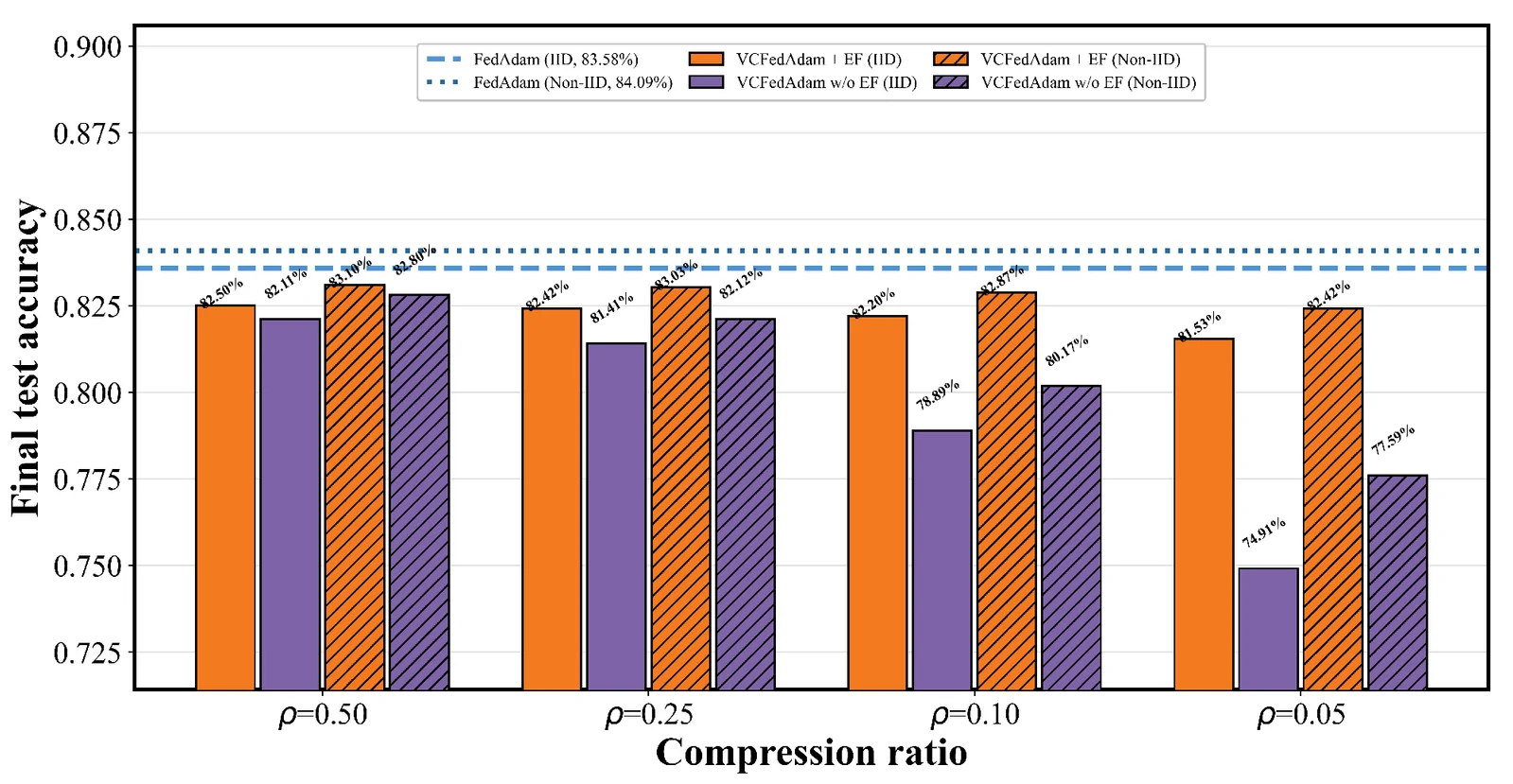
MNIST accuracy under different compression ratios and data distributions.

The final MNIST accuracy under different compression ratios is presented in Figure 13. Competitive accuracy is preserved under both IID and non-IID settings when error feedback is enabled. As the sketch dimension is reduced, a moderate accuracy decrease is observed. A substantially larger degradation is produced when error feedback is disabled, especially under aggressive compression. The importance of residual correction for preserving model utility is therefore demonstrated.

Notably, the small utility gap under both IID and non-IID settings is mainly caused by the approximation introduced during sketch compression. At ρ=0.25, the accuracy losses are limited to 0.64 percentage points on CIFAR-10 and 1.16 percentage points on MNIST. Error feedback compensates for discarded update information across communication rounds. A larger degradation is observed only when stronger compression is applied or error feedback is disabled.

## 8. Conclusions

In this paper, VCFedAdam was proposed for verifiable and compressed FL in DSNs. By performing masking, aggregation, and verification in the sketch domain, VCFedAdam reduces the overhead of full-dimensional SA while protecting individual sensing-model updates. ANs release only aggregate materials for the fixed online set, enabling dropout-aware mask recovery and lightweight user-side verification through scalar sketch tags. Security analysis shows that the scheme preserves update privacy and detects incorrect aggregate sketches. Experimental results demonstrate that VCFedAdam reduces computation, communication, and storage overhead while maintaining competitive accuracy under different compression ratios and dropout rates. VCFedAdam is better suited for edge scenarios with limited communication resources, as compression can significantly reduce transmission overhead. However, on devices with limited computing power but ample network bandwidth, the additional computation required for sketch encoding may diminish this advantage. Therefore, in practical deployments, an appropriate compression ratio should be selected based on the device’s computing capabilities and network conditions. Future work will involve further evaluating its latency and energy consumption on MCUs, Raspberry Pis, and wearable devices.

## Figures and Tables

**Table 1:** Comprehensive Comparison of Aggregation Schemes in FL.

Reference	Year	Approach	Key Techniques	Supported Functions	Fault Tolerance
Privacy-Preserving Aggregation					
Bonawitz et al. [20]	2017	SA	SA; SS	Private summation	SS-based dropout recovery
Bouamama et al. [21]	2023	EdgeSA	ENC; SS; AUTH	Privacy protection; integrity checking	No
Fotohi et al. [22]	2024	FEDANIL+	ENC; COMP; BC	Privacy protection; update compression	BC-assisted coordination
Yu et al. [23]	2025	PrivLDFL	ENC; COMP	Privacy protection; dynamic participation	Partition-based dropout handling
He and Yu [24]	2025	SWAS	ENC; VER	Weighted aggregation; data-size protection	No
Ghazal et al. [25]	2025	GFL	AUTH; ENC	Privacy protection; data integrity	No
Verifiable Aggregation					
Xu et al. [16]	2019	VerifyNet	SA; VER	Privacy protection; result verification	SS-based dropout recovery
Guo et al. [26]	2020	VeriFL	VER; COM	Result verification; efficient communication	SA-compatible dropout handling
Eltaras et al. [27]	2023	EVP	AUX; SA; VER	Private aggregation; user-side verification	AUX-assisted mask recovery
Xu et al. [17]	2025	EVSA	AUX; SS; MAC	Private aggregation; lightweight verification	AUX-assisted SS recovery
Xie et al. [13]	2025	VCFL	SA; COM; VER	Dynamic verification; collusion resistance	Dynamic verification
Bouamama et al. [28]	2025	VeSAFL	AUX; SS; AUTH	Authentication; VA	Threshold SS and multi-aggregator support
Proposed Scheme					
Ours	–	Sketch-VA	COMP; SA; AUX; VER; OPT	Compressed aggregation; lightweight verification; adaptive update	Fixed online set and aggregate mask recovery

*Note:* SA = secure aggregation, SS = secret sharing, ENC = encryption-based protection, COMP = compression, VER = verification, COM = commitment, AUX = auxiliary or compute-node assistance, AUTH = authentication, BC = blockchain, MAC = message authentication code, OPT = adaptive optimization, VA = verifiable aggregation.

**Table 2:** Computation complexity comparison.

Scheme	User-Side Cost	Auxiliary-Node Cost	Server-Side Cost
EVP [27]	$O(NMd+NMC_{\mathrm{DH}})$	$O(NMd+NMC_{\mathrm{DH}})$	O(Nd+Md)
EVSA [17]	$O(Nd+NMC_{\mathrm{DH}})$	$O(NM+NMC_{\mathrm{DH}})$	$O(Nd+M^{2})$
VeSAFL [28]	$O(NMd+NMC_{\exp})$	$O(NMd+NMC_{\mathrm{pair}})$	$O(Md+MC_{\mathrm{pair}})$
VCFL [13]	$O(N^{2}d+NC_{\exp})$	–	O((N−|R|)|R|d+Nd)
VCFedAdam	$O(Nd+NMr+NMC_{\mathrm{DH}})$	O(NM)	O(|R|r+d)

**Table 3:** Comparative phase-level computation overhead.

Dropout	Phase	EVP [27]		EVSA [17]		VeSAFL [28]		VCFL [13]		VCFedAdam	
		*N* = 100	*N* = 500	*N* = 100	*N* = 500	*N* = 100	*N* = 500	*N* = 100	*N* = 500	*N* = 100	*N* = 500
0%	Sharing Key	137.17 ms	711.85 ms	49.49 ms	306.55 ms	N/A	N/A	768.63 ms	23,167.55 ms	53.21 ms	288.55 ms
	Masking Input	117.67 ms	705.59 ms	33.19 ms	213.69 ms	99.34 ms	552.22 ms	374.18 ms	12,435.30 ms	73.70 ms	443.83 ms
	Unmasking Input and aggregation	108.35 ms	697.66 ms	43.29 ms	238.48 ms	5.19 ms	22.51 ms	0.47 ms	1.59 ms	1.63 ms	6.63 ms
	Verification	N/A	N/A	N/A	N/A	64.17 ms	340.30 ms	4.75 ms	43.19 ms	16.78 ms	84.14 ms
10%	Sharing Key	167.94 ms	831.39 ms	60.50 ms	282.82 ms	N/A	N/A	948.10 ms	23,891.04 ms	57.25 ms	305.19 ms
	Masking Input	148.13 ms	712.66 ms	42.88 ms	208.46 ms	105.98 ms	517.84 ms	533.24 ms	13,165.54 ms	81.66 ms	480.66 ms
	Unmasking Input and aggregation	139.64 ms	640.91 ms	46.12 ms	222.55 ms	6.60 ms	21.24 ms	44.78 ms	1174.06 ms	1.54 ms	7.42 ms
	Verification	N/A	N/A	N/A	N/A	72.12 ms	350.73 ms	4.39 ms	39.55 ms	15.37 ms	75.46 ms
20%	Sharing Key	177.10 ms	887.58 ms	64.88 ms	318.27 ms	N/A	N/A	986.69 ms	24,412.39 ms	62.34 ms	275.73 ms
	Masking Input	165.88 ms	818.30 ms	50.90 ms	240.09 ms	124.24 ms	624.25 ms	555.01 ms	13,023.94 ms	94.04 ms	433.65 ms
	Unmasking Input and aggregation	126.54 ms	619.95 ms	47.08 ms	220.74 ms	7.04 ms	35.00 ms	84.39 ms	1973.44 ms	2.02 ms	5.75 ms
	Verification	N/A	N/A	N/A	N/A	79.39 ms	397.06 ms	4.00 ms	34.53 ms	13.50 ms	67.48 ms

**Table 4:** Communication complexity comparison.

Scheme	Total Communication Complexity
EVP [27]	O(Nd+Md+NMκ)
EVSA [17]	O(Nd+NMκ)
VeSAFL [28]	$O(NMd+M^{2}d+N\kappa )$
VCFL [13]	$O(Nd+N^{2}\kappa )$
VCFedAdam	O(|R|k+NMκ)

**Table 5:** Ablation study of the main components in VCFedAdam.

Variant	Sketch	RC	CBV	ANs	Comp. (ms)	Comm. (MB)
FedAdam + Traditional SA	No	No	No	–	1371.72	17.989
VCFedAdam-FD	No	N/A	Yes	5	162.75	16.428
VCFedAdam w/o RC	Yes	No	Yes	5	133.47	4.428
VCFedAdam w/o CBV	Yes	Yes	No	5	111.19	2.074
VCFedAdam (M=1)	Yes	Yes	Yes	1	42.60	4.105
VCFedAdam (ρ=0.25)	Yes	Yes	Yes	5	133.79	4.428
VCFedAdam (ρ=0.10)	Yes	Yes	Yes	5	127.94	2.024
VCFedAdam (ρ=0.05)	Yes	Yes	Yes	5	122.37	1.217

**Table 6:** Storage overhead of the proposed protocol.

Entity	Stored Items	Asymptotic Storage	Concrete Storage
User	*M* seeds, local residual vector	O(Mκ+dlogq)	80.16 KB
AN	User seeds and round scalars	O(Nκ)	3.33 KB
Server	Public keys, aggregate sketch, Adam states	O((N+M)κ+rlogq+3dlogq)	266.72 KB
Overall	All users, ANs, and server	O(Nd+Mr+NMκ)	8.30 MB

## Data Availability

The datasets used in this study are publicly available. The MNIST dataset is available from the UCI Machine Learning Repository at https://archive.ics.uci.edu/dataset/683/mnist+database+of+handwritten+digits (accessed on 7 July 2026). The CIFAR-10 dataset is available from the UCI Machine Learning Repository at https://archive.ics.uci.edu/dataset/691/cifar+10 (accessed on 7 July 2026).

## Appendix A. Main Notations Used in VCFedAdam

The main notations used throughout VCFedAdam are summarized in Table A1.

**Table A1:** Main notations used in VCFedAdam.

Symbol	Description	Symbol	Description
U	Set of all users	A	Set of auxiliary nodes
$U_{i}$	User *i*	$A_{m}$	Auxiliary node *m*
S	Aggregation server	*R*	Fixed online user set
|R|	Number of online users	$N_{\min}$	Minimum required online users
$n_{i}$	Aggregation weight of $U_{i}$	$N_{R}$	Total weight $\sum_{U_{i}\in R}n_{i}$
*d*	Original model dimension	*r*	Sketch dimension
ρ	Compression ratio r/d	*q*	Prime modulus
κ	Cryptographic security parameter	$\Gamma_{R}^{\left(t\right)}$	Aggregation state for *R* in round *t*
$W_{i}^{\left(t\right)}$	Quantized local update	$e_{i}^{\left(t\right)}$	Error-feedback residual
$u_{i}^{\left(t\right)}$	Residual-corrected weighted update	$S_{t}$	Sketch matrix in round *t*
$y_{i}^{\left(t\right)}$	Local compressed sketch	${\hat{y}}_{i}^{\left(t\right)}$	Masked local sketch
$Y_{R}^{\left(t\right)}$	Aggregate sketch for *R*	$\mu_{i,m}^{\left(t\right)}$	Mask component from $A_{m}$
$\mu_{i}^{\left(t\right)}$	Total sketch mask	$s_{i,m}^{\left(t\right)}$	Shared round seed
$a_{i,m}^{\left(t\right)}$	Sketch-masking key	$k_{i,m}^{\left(t\right)}$	MAC key
$A_{R,m}^{\left(t\right)}$	Aggregate mask key for *R*	$K_{R,m}^{\left(t\right)}$	Aggregate MAC key for *R*
$C_{R}^{\left(t\right)}$	Aggregation-state commitment	α	Aggregated scalar challenge
ξ	Aggregated CBV random seed	$c_{t}$	CBV challenge vector
$\theta_{i}^{\left(t\right)}$	User verification tag	$\Theta^{\left(t\right)}$	Aggregate verification tag
$D_{t}\left(\cdot \right)$	Sketch-decoding function	$\lambda_{s}$	Sketch normalization factor
$m_{t}$	First-order FedAdam moment	$v_{t}$	Second-order FedAdam moment
